# USP32 confers cancer cell resistance to YM155 via promoting ER-associated degradation of solute carrier protein SLC35F2

**DOI:** 10.7150/thno.63806

**Published:** 2021-09-27

**Authors:** Arun Pandian Chandrasekaran, Kamini Kaushal, Chang-Hwan Park, Kye-Seong Kim, Suresh Ramakrishna

**Affiliations:** 1 Graduate School of Biomedical Science and Engineering, Hanyang University, Seoul, South Korea.; 2 College of Medicine, Hanyang University, Seoul, South Korea.

**Keywords:** DNA damage, dose response, human tumor tissues, cell apoptosis, drug transport-cargo

## Abstract

**Background:** The most commonly preferred chemotherapeutic agents to treat cancers are small-molecule drugs. However, the differential sensitivity of various cancer cells to small molecules and untargeted delivery narrow the range of potential therapeutic applications. The mechanisms responsible for drug resistance in a variety of cancer cells are also largely unknown. Several deubiquitinating enzymes (DUBs) are the main determinants of drug resistance in cancer cells.

**Methods:** We used CRISPR-Cas9 to perform genome-scale knockout of the entire set of genes encoding ubiquitin-specific proteases (USPs) and systematically screened for DUBs resistant to the clinically evaluated anticancer compound YM155. A series of *in vitro* and *in vivo* experiments were conducted to reveal the relationship between USP32 and SLC35F2 on YM155-mediated DNA damage in cancer cells.

**Results:** CRISPR-based dual-screening method identified USP32 as a novel DUB that governs resistance for uptake of YM155 by destabilizing protein levels of SLC35F2, a solute-carrier protein essential for the uptake of YM155. The expression of USP32 and SLC35F2 was negatively correlated across a panel of tested cancer cell lines. YM155-resistant cancer cells in particular exhibited elevated expression of USP32 and low expression of SLC35F2.

**Conclusion:** Collectively, our DUB-screening strategy revealed a resistance mechanism governed by USP32 associated with YM155 resistance in breast cancers, one that presents an attractive molecular target for anti-cancer therapies. Targeted genome knockout verified that USP32 is the main determinant of SLC35F2 protein stability *in vitro* and *in vivo*, suggesting a novel way to treat tumors resistant to small-molecule drugs.

## Introduction

Despite advances in cancer therapies, cancer continues to cause millions of deaths worldwide. Standard chemotherapeutic treatments such as small molecules have not been successful 1,2. Increasing a drug's concentration is not effective as dosages in many cases have reached maximum tolerable levels, and further increases could damage tissues or organs. Targeted, patient-specific, or precision therapies have been proposed, but outcomes to date have proved discouraging. Understanding the transport-cargo profile of potential drugs might help resolve disputes regarding transporter-mediated resistance mechanisms acquired by cancer cells.

YM155 is a small molecule that targets various cancers 3-6. Despite positive results from clinical trials for non-small-cell lung cancer, cellular response to YM155 is slow and incomplete in other cancer types 7. Although the exact mode of action remains unknown, one study reported that YM155 downregulates inhibition of the apoptosis protein (IAP) survivin through ILF3/NF110 binding 8. Survivin belongs to the IAP protein family and has been linked to both cell survival and mitotic control in cancer 9-11. Because there is a link between high survivin expression in tumors and poor recovery in patients with various malignancies, survivin is being explored as a potential new target in cancer treatments 12,13. Recent research has revealed an essential role for the expression of SLC35F2, a solute carrier protein for YM155 uptake, which leads to DNA damage 14. The relative endogenous expression level of SLC35F2 in several cancer cells is the sole determinant of cellular uptake of YM155, and the uptake of YM155 reportedly was reduced in cancer cells that express low level of SLC35F2 14. Identifying the factors responsible for preventing the expression of SLC35F2 in YM155-resistant cancer cells is critical to improving the clinical efficacy of small-molecule-mediated cancer therapeutics.

Post-translational modifications, particularly ubiquitination and deubiquitination, play a vital role in the maintenance of protein turnover 15,16. Studies have suggested that several deubiquitinating enzymes (DUBs) are involved in drug resistance in cancer cells 17,18. Overexpression of DUBs can negatively affect small-molecule inhibitor efficacy and decrease the therapeutic value of the drugs 19-21. To shed light on the mechanism of YM155 resistance, and improve its therapeutic value, we used our recently developed genome-scale CRISPR-based DUB knockout library kit to identify DUBs that may cause resistance to cellular uptake of YM155 22.

Our screening process allowed us to identify several DUBs regulating the expression of the SLC35F2 protein. We determined that USP32, a membrane protein, can confer resistance to cellular uptake of YM155 by destabilizing endogenous expression of SLC35F2 in cancer cells. The loss of USP32 function induced DNA damage and apoptosis by enriching the cellular uptake of YM155 both *in vitro* and *in vivo*. The results imply that USP32 inhibition in cancer cells resistant to YM155 due to reduced SLC35F2 expression may be an effective YM155-based cancer treatment.

## Materials and Methods

### Cell culture, chemical treatment, and plasmids

HEK293, HeLa, PC3, SW480, SW620, HCT116, HCC1419, HT29, MDAMB231, A549, MG63, MCF7, and BT474 (ATCC, VA, USA) cells were cultured in Dulbecco's modified Eagle medium (DMEM; Gibco, CA, USA) supplemented with 10% fetal bovine serum (FBS; Gibco) and 1% penicillin and streptomycin (Gibco) at 37 °C in a humidified atmosphere with 5% CO_2_. Cycloheximide, MG132, and Chloroquine were purchased from Sigma-Aldrich (MO, USA). YM155 was purchased from Selleckchem (TX, USA) and Eeyarestatin I was purchased from Tocris. Flag-SLC35F2 was received from Dr. Giulio Superti-Furga. Flag-SLC35F2 was then subcloned into a pcDNA 3.1 6XMyc-vector. HA-USP32 WT and HA-USP32 CA were received from Dr. Jacques Neefjes. HA-Ubiquitin (Addgene #18712) was procured from Addgene (MA, USA). Cas9-2A-mRFP-2A-PAC was purchased from Toolgen (Seoul, South Korea).

### Transfection

Experiments with HEK293 and HeLa cells were transfected with Lipofectamine 2000 (Thermo Fisher, MA, USA) followed the manufacturer's recommendations. Experiments with MCF7 and BT474 cells were transfected with the indicated plasmids using Lipofectamine 3000 (Thermo Fisher, MA, USA) and following the manufacturer's recommendations. The following day, puromycin (Gibco) (1.5 µg/mL for MCF7 and 1 µg/mL for BT474 cells) was selected 48 h prior to analysis.

### Cas9 and sgRNA constructs

Plasmid-encoding Cas9-2A-mRFP-2A-PAC (puromycin N-acetyl-transferase, a puromycin resistance gene) was purchased from Toolgen (Seoul, South Korea). The sgRNAs targeting USP32 used in this study were obtained from our DUB-knockout library kit 22. Briefly, oligonucleotides were synthesized (Bioneer, Seoul, South Korea) and annealed, and T4 polynucleotide kinase was then used to add terminal phosphates to the annealed oligonucleotides (Biorad, CA, USA). As a mock control, non-targeted sgRNAs (scrambled sgRNA sequences) were designed as forward 5′ GCCACGCGTAAGCGGTCCGC 3′ and reverse 5′ GCGGACCGCTTACGCGTGGC 3′. The vector was digested with the *BsaI* restriction enzyme and ligated with the annealed oligonucleotides.

### Cell viability assay

For the initial screening of DUBs using a cell viability assay, we co-transfected HeLa cells with sgRNA targeting 50 DUBs and a Cas9 plasmid containing the puromycin resistance gene. As a mock control, scrambled non-targeted sgRNAs sequences were designed. One day later, transfected cells were selected with puromycin (2 µg/mL) to improve transfection efficiency. Selected cells were then seeded in 96-well plates for the assay. Cells were treated with either dimethyl sulfoxide (DMSO) or indicated concentrations of YM155. After 24 h, either CellTiter-Glo (Promega, WI, USA) or CCK-8 assay reagent (Dojindo Molecular Technologies, MD, USA) was added to each well following the manufacturers' protocols. Luminescence or absorbance was measured and values were recorded graphically.

### Western blot analysis and antibodies

To screen functional DUBs that regulate SLC35F2 protein, we co-transfected HeLa cells with sgRNA targeting 50 DUBs and Cas9 plasmids. The cells were washed with phosphate-buffered saline (PBS) (PAN Biotech, Aidenbach, Germany), harvested, lysed with a lysis buffer, and incubated on ice for 20 min. The samples were then centrifuged and the resulting supernatants collected and kept in separate tubes. A Bradford assay (Biorad, CA, USA) was used to estimate protein concentrations. Western blots were performed using SLC35F2-specific antibodies (25526-1-AP; 1:1000) from Proteintech (IL, USA). The antibodies used in our experiments were as follows: γH2AX (05-636; 1:1,000) from Millipore (MA, USA); SLC35F2 (NBP1-59890; 1:1,000) from Novus (CO, USA); USP19 (25768-1-AP; 1:1000) and USP49 (18066-1-AP; 1:1,000) from Proteintech; H2AX (sc-517336; 1:1000), SLC35F3 (sc-515378; 1:1000), USP32 (sc-374465; 1:1,000), GAPDH (sc-47724; 1:1,000), Myc (sc-40; 1:1,000), and Calnexin (sc-23954, 1:100) from Santa Cruz Biotechnology (TX, USA); and Flag (M185-3L; 1:1,000) from MBL International (MA, USA), and AKT (9272, 1:1000), p-AKT (9271, 1:1000), PI3K (4292, 1:1000), p-PI3K (4228, 1:1000), mTOR (2983, 1:1000), p-mTOR (2971, 1:1000) and Survivin (2808, 1:1000) were procured from Cell Signaling Technology (MA, USA).

### Generation of single cell-derived USP32 knockout clones in MCF7 and BT474 cells

Single cell-derived stable knockout clones of USP32 were generated in MCF7 and BT474 cells. MCF7 or BT474 cells were co-transfected with sgRNA2 targeting USP32 and Cas9 at a 1:2 weight ratio using Lipofectamine 3000. One day after transfection, the cells were selected with puromycin (1.5 µg/mL for MCF7 and 1 µg/mL for BT474 cells) for 48 h. A small portion of transfected cells were harvested to validate sgRNA efficiency using a T7E1 assay, and band intensity (indel %) was analyzed using ImageJ software. The sample with higher indel frequency was used to generate stable knockout clones. Cells were seeded at a density of 0.25 per well in 96-well plates. After 12 to 16 days, each well was analyzed for a single cell with rounded morphology and re-plated into 24-well plates for expansion. Once cells reached 70% confluence, a small portion of the cells was harvested to isolate genomic DNA and further subjected to a T7E1 assay to identify the knockout clones. Samples with expected cleaved bands were marked as T7E1-positive clones and expanded for further analysis. Before performing experiments, knockout clones were placed in a liquid-nitrogen tank for long-term storage. Knockout clones were validated by western blots, and gene disruption was confirmed by Sanger sequencing.

### T7E1 assay

A T7E1 assay was conducted following a previously described protocol 23. We isolated genomic DNA (Promega, WI, USA) from the collected samples following the manufacturer's instructions. A polymerase chain reaction (PCR) were used to amplify the region containing the nuclease target site using the following hemi-nested primers. USP32 first PCR: forward 5′ AGATGGAAGTGGAGTTTCTGGA 3′ and reverse 5′ GTCACAGATGGCTCAAGGCTA 3′; USP32 second PCR: 5ʹ AGATGGAAGTGGAGTTTCTGGA 3′ and reverse 5′ ACATGAGCACTGTTTCAGGTTC 3′. The PCR amplicons were denatured at 95 °C and annealed to form heteroduplex DNA and treated with 5 units of T7E1 enzyme (New England Biolabs, MA, USA) at 37 °C for 20 min. Gel electrophoresis was performed using 2% agarose to separate cleaved regions. ImageJ was used to quantify the mutation frequencies using the following equation: mutation frequency (%) = 100 × (1 - [1 - fraction cleaved]^1/2^), where the fraction cleaved was the total relative density of the cleavage bands divided by the sum of the relative density of cleavage and uncut bands. Potential off-target sites were selected from CRISPOR 24 and validated using a T7E1 assay.

### Immunoprecipitation

Cells were washed with PBS, harvested and lysed in an immunoprecipitation (IP) buffer containing 50 mM Tris-HCl (pH 8.0), 150 mM NaCl, 10 mM KCl, 1.5 mM MgCl_2_, 0.5% NP-40, 10% glycerol, and 1 mM EDTA, and then incubated for 20 min. Centrifugation was used to separate the supernatant. The IP was carried out by incubating lysates with the indicated antibodies at 4 °C overnight followed by the addition of 20 µL of protein A/G Sepharose beads (Santa Cruz Biotechnology) at 4 °C for 2-3 h. The beads were then washed three times with an IP lysis buffer and eluted with a sodium dodecyl sulfate (SDS) sample buffer. Endogenous USP32 or SLC35F2 immunoprecipitation was performed in HeLa cells using 3 µg of USP32 or 3 µg of SLC35F2 antibodies at 4 °C followed by a western blot with relevant antibodies. For the ubiquitination assay, transfected cells were treated with or without MG132 (10 µM) for 4 h and harvested. The IP was then performed, followed by western blotting with the indicated antibodies. TrueBlot secondary antibodies (Rockland Antibodies, cat 18-8816-31) was used to prevent interference from heavy and light immunoglobulin chains in the IP assays.

### Immunofluorescence

PC3, SW480, HeLa, HEK293, MCF7, or BT474 cells were either transfected with the indicated plasmids or incubated with or without YM155. The cells were then fixed for 15 min in 4% paraformaldehyde at room temperature. After washing twice with PBS, cells were permeabilized in PBS containing 0.1% Triton for 5 min, washed extensively in PBS, and blocked with 3% bovine serum albumin (BSA). Cells were then incubated with the indicated primary antibodies diluted in BSA at 4 °C overnight. The next day, the cells were washed and incubated with appropriate Alexa Fluor 488/594-conjugated secondary antibodies (Invitrogen, CA, USA) for 1 h in the dark. Cells were washed three times with PBS in the dark and incubated with DAPI (Invitrogen) followed by mounting using VectaShield (Vector Laboratories, CA, USA) on glass slides. Cells were visualized and images were taken using a Leica fluorescence microscope (Leica, DM 5000B; Leica CTR 5000; Wetzlar, Germany).

### Duolink proximity ligation assay

The Duolink *in situ* proximity ligation assay (PLA) kit was used to identify the interaction between USP32 and SLC35F2 (DUO92101, Sigma Aldrich). HeLa cells were fixed for 10 min at room temperature in 4% paraformaldehyde and then blocked with 1× blocking solution. The cells were treated overnight at 4 °C with primary antibodies targeting USP32 and SLC35F2, followed by 1 h at 37 °C with PLA probes. The ligation-ligase solution was added after three washes and incubated for 30 min at 37 °C. The slides were incubated in an amplified polymerase solution for 100 min at 37 °C in the dark. Subsequently, cells were stained with calnexin antibody to stain the endoplasmic reticulum. Finally, the cells were stained with DAPI-containing mounting medium. A Leica fluorescence microscope was used to capture the fluorescence images (Leica, DM 5000B; Leica CTR 5000; Wetzlar, Germany).

### Tandem ubiquitin-binding entities assay

The ubiquitination status of SLC35F2 was determined by a tandem ubiquitin binding entities (TUBEs) assay (LifeSensors, PA, USA). HeLa cells were treated with DMSO or 5 ng/mL of interleukin (IL)-1β for the indicated time intervals. The HeLa cells were then harvested for protein extraction following the manufacturer's protocol. Briefly, equilibrated TUBEs were added to the required amount of protein lysate and incubated at 4 °C for 3 h. Centrifugation was the performed at a low rpm to collect the beads and washed twice with TBS-T. During the final wash, a small volume of supernatant was left to avoid damage. An SDS sample buffer was added, followed by boiling for 5 min and centrifuging at a high rpm before performing SDS-polyacrylamide gel electrophoresis (PAGE) analysis. Finally, immunoblotting with SLC35F2 antibodies was performed to determine the ubiquitination status.

### RT-qPCR analysis

HeLa cells were treated with indicated concentrations of MG132 for 6 h and harvested for RNA extraction. We extracted RNA using the TRIzol reagent (Favorgen, Pingtung, Taiwan). RNase-free water (40 mL) was added to the samples to reconstitute the RNA, which was quantified using a Nanodrop (Thermo Fisher Scientific). We synthesized cDNA using the SuperScript III First-Strand Synthesis System (Thermo Fisher Scientific) following the manufacturer's protocol. Briefly, 1 µg of total RNA was reverse-transcribed with a 25 pmol oligo-dT primer and a 50 pmol random hexamer using the SuperScript III kit. The cDNA was then diluted at 1:10 with a dilution buffer included in the kit. Reverse-transcription quantitative PCR (RT-qPCR) was performed using a real-time PCR system (Thermo Fisher Scientific) and Fast SYBR Green Master Mix (Thermo Fisher Scientific) with the following primers: SLC35F2 forward, 5′-GTGAGGAATACATCGTGAA-3′ and reverse, 5′-CAAACAGAAAGAGTCCAACA-3′; and GAPDH forward, 5′-CTGACTTCAACAGCGACACC-3′ and reverse, 5′-TAGCCAAATTCGTTGTCATACC-3′. We determined the relative quantification of gene expression using the 2-ΔΔCq method 25. The PCR conditions were: pre-incubation at 95 °C for 3 min followed by 45 cycles of 95 °C for 15 s and 60 °C for 60 s. The final amplification cycle was followed by a melt-curve analysis for the specificity of the RT-qPCR.

### LC-MS/MS analysis

Intracellular YM155 concentrations were quantified using a TSQ Quantiva Triple-Stage Quadrupole Mass Spectrometer (Thermo Fisher Scientific) with electrospray ionization in positive-ion, multiple-reaction monitoring (MRM) mode. The settings of the mass spectrometer were: method duration - 15 min, ion source type - H-ESI, spray voltage (positive ion) - 3.5 kV, spray voltage (negative ion) - 2.5 kV, sheath gas - 40 arbitrary units, aux gas - 12 arbitrary units, sweep gas - 1 arbitrary unit, ion transfer tube temperature - 333 °C, and vaporizer temperature - 317 °C. The MRM *m/z* transitions monitored were: *m/z* 363.183 for precursor and 93.071-305 for product with a dwell time of 198.37 ms. Data were acquired in MRM mode using a resolution of 0.7 full-width at half-maximum for both quadrupoles 1 and 3 with a 0.600 s cycle time.

### Ethidium homodimer-1 staining

The total dead populations were estimated by staining the cells with ethidium homodimer-1 (EthD-1) (Thermo Fisher). Briefly, PC3 or SW480 cells were transfected with indicated plasmids. At 48 h post-transfection, 10 nM of YM155 was added to the transfected cells and incubated for another 24 h. Then, 150 µL of PBS containing 4 µM EthD-1 was added to the transfected cells, which were incubated for 30 min at room temperature. Images were captured using an inverted fluorescence microscope (IX71, Olympus, Tokyo, Japan).

### Apoptosis assay

Cellular apoptosis was detected by measuring annexin V/7-AAD populations using a BD FACSCanto II flow cytometer (BD Biosciences, CA, USA). Briefly, MCF7 and BT474 cells (wild type, USP32KO and reconstituted with USP32) were treated with either DMSO or indicated concentrations of YM155 for 24 h. Cells were harvested and washed twice with PBS containing 10% FBS. A binding solution (5 µL of annexin V and 5 µL of 7-AAD, BD Biosciences) was added to the cells and kept in the dark for 15 min. Flow cytometry was performed within 1 h. For propidium iodide (PI) staining (BD Biosciences), the mentioned cell lines were treated with DMSO or indicated concentrations of YM155 for 24 h. Cells were then harvested, washed twice with ice-cold PBS containing 10% FBS and fixed with 70% ethanol until use. Next, 2 mg/mL of RNaseA was added to the cells at 4 °C for 15 min followed by 10 µL of PI at a concentration of 50 mg/mL at room temperature for 10 min. Finally, DNA content was measured using flow cytometry. Data were analyzed using FACSDiva software (version 8; BD Biosciences).

### 14-d colony formation assay

MCF7, MCF7_USP32KO, MCF7KO_ReconWT, MCF7KO_ReconCA, BT474, BT474_USP32KO, BT474KO_ReconWT, and BT474KO_ReconCA cells were used for a formation assay. Complete DMEM medium (1×) and agarose (1%) were mixed at a ratio of 1:1 (v/v) and plated onto 35 mm culture plates. The cells were seeded the next day at a density of 1 × 10^4^ per well mixed with 0.7% of DMEM containing agarose. Then, 1% DMSO or 5 nM YM155 was added to the complete DMEM. The aforementioned drug was added to the medium every other day for 14 days. Crystal violet dye (0.01%) diluted in 20% methanol was added for 5 min at room temperature to stain the anchorage-independent colonies. A light microscope was used to count the colonies at 4× magnification.

### Transwell cell migration and invasion assays

Cellular migration and invasion were assessed using 0.8 µm Transwell chambers coated with Matrigel (only for invasion assays) for 1 h at 37°C (Corning, NY, USA) according to the manufacturer's instructions. Briefly, the mentioned groups at a density of 3.0 x 10^4^ cells per well were suspended in 500 µL of serum-free DMEM medium and placed into 24-well plates. Next, 750 µL of complete medium was added and incubated 37 °C with 5% CO_2_. The following day, we scraped off the cells on the top surface of the insert and the cells on the bottom surface were fixed with ice-cold methanol, with crystal violet used for staining. The cell number was counted using light microscopy and presented graphically.

### Xenograft tumor experiment

Female NOD scid γ (NSG) mice (6 weeks old) were used for *in vivo* experiments. The animal study was approved by the IACUC. All mice were maintained in standard conditions with a 12 h light/dark cycle and given access to food and water. Mock MCF7 cells or USP32-knockedout MCF7 cells (4.0 x10^6^) in DMEM:Matrigel (1:1) (BD Biosciences) were subcutaneously injected into the right flank of each mouse. To calculate the tumor volume, we used the formula V = D × d^2^ × 0.5, where D is the long axis, and d the short axis of the tumor. Once the tumor volume reached approximately 75 mm^3^, we randomized the mice into groups (n = 5) and injected saline or YM155 (7.5 mg/kg) intraperitoneally twice in a week. At the end of the study, all mice were sacrificed by CO_2_ asphyxiation. Tumors were dissected from the mice, images were taken, and the tumors were subjected to immunohistochemistry analysis.

### Immunohistochemistry

For tissue microarray analysis, we purchased breast (n = 21), colon (n = 32) and lung (n = 32) cancer tissues from ISU Abxis (Gyeonggi-do, South Korea). Formalin-fixed, paraffin-embedded tissue samples were processed and incubated with USP32 (sc-376491, 1:100) from Santa Cruz Biotechnology or SLC35F2 (NBP1-59890, 1:100) from Novus Biologics following the manufacturers' recommendations. These samples were counterstained with hematoxylin, dehydrated, and mounted.

Tumor-tissue samples obtained from the mice were fixed with 4% paraformaldehyde and embedded in paraffin. Thin sections (5 µm) were stained with USP32 (sc-376491, 1:100) from Santa Cruz Biotechnology, SLC35F2 (NBP1-59890, 1:100) from Novus Biologics, γH2AX (05-636, 1:250) from Millipore, and cleaved PARP (1:100) from Cell Signaling Technology following the manufacturers' recommendations. All images were taken using a Leica DM5000 B microscope (Leica).

### Statistical analysis

All results were recorded as means and standard deviations from at least three independent experiments (unless otherwise stated in figure legends). Comparisons between two groups were analyzed using the Student's *t* test. Experiments involving three or more groups were analyzed by one-way or two-way analysis of variance (ANOVA) followed by the Tukey's post hoc test. A *P* value < 0.05 was considered statistically significant. All statistical analyses were performed in GraphPad Prism 9 software (CA, USA).

## Results

### CRISPR-based genome-scale screening of USP family proteins exhibiting drug resistance to YM155 treatment

**Figure 1:**
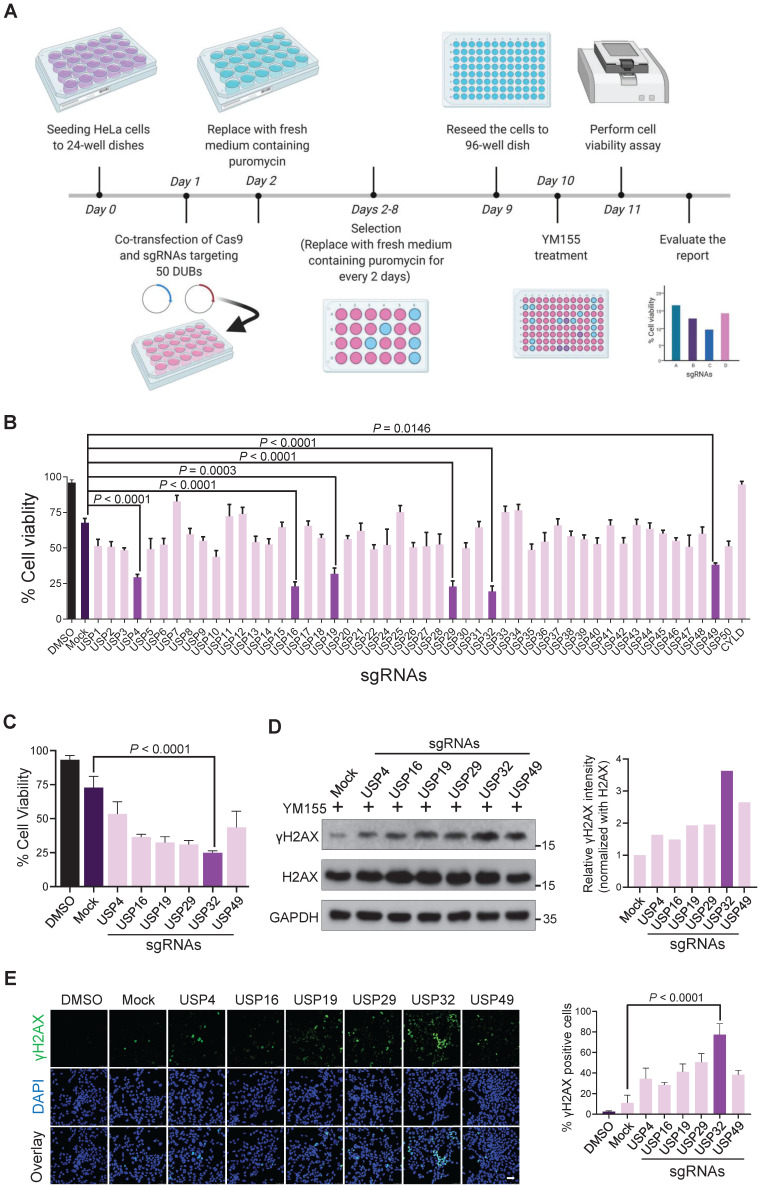
** CRISPR-based genome-scale screening of USPs family proteins showing drug resistance to YM155 treatment through cell viability assay. (A)** Schematic of a screening system based on DUB knockout and YM155 treatment. Day 0: HeLa cells were seeded and maintained in DMEM. Day 1: A DUB-knockout library kit consisting of sgRNAs individually targeting an entire set of genes encoding USPs along with Cas9 were co-transfected using the Lipofectamine 2000 in HeLa cells. Day 2: Complete medium containing puromycin (2 µg/mL) was replaced. Day 2-8: Cells were grown under puromycin selection for a week. Day 9: HeLa cells were re-seeded to 96-well plates at a density of 10,000 cells/well for a cell viability assay. Day 10 and 11: 25 nM YM155 was treated and incubated for 24 h and subjected to the cell viability assay. **(B)** The percentage of cell viability was measured from (A) and plotted as a bar graph. HeLa cells treated with DMSO served as a negative control. HeLa cells co-transfected with scrambled sgRNA and Cas9 and then treated with YM155 served as mock control. **(C)** Cell viability of the top-ranking candidates. **(D)** HeLa cells were transfected with the top-ranking candidates and treated with 25 nM YM155 for 24 h and subjected to western blotting with γH2AX antibodies. H2AX and GAPDH were used as loading controls. Relative expression of γH2AX was quantified using ImageJ software (right panel). **(E)** Immunofluorescence images showing γH2AX foci formation in HeLa cells co-transfected with the top-ranking candidates and treated with 25 nM YM155 for 24 h. γH2AX positive cells were quantified and represented as a bar graph (right panel). **(B, C and E)** Data are presented as the mean and standard deviation of three independent experiments. One-way ANOVA followed by Tukey's post hoc test was used with the indicated *P* value. Scale bar = 50 µm.

Genome-wide screening for DUBs rescued from resistance to YM155-induced cytotoxicity in cancer cells was carried out. A CRISPR-Cas9-based DUB-knockout library kit consisting of sgRNAs individually targeting entire USPs and Cas9 were co-transfected into HeLa cells 22. Then, 25 nM YM155 was treated and subjected to cell viability assay (Figure 1A). The sgRNAs targeting USP4, USP16, USP19, USP29, USP32, and USP49 displayed more sensitivity to YM155 (Figure 1B). Among other candidates, USP32 knockout was more sensitive to YM155 (Figure 1C).

YM155 induces DNA double-strand breaks (DSBs) in cancer cells 14. We hypothesized that knockout of top-ranking candidates can improve YM155 treatment. Thus, we treated 25 nM YM155 and analyzed γH2AX expression, a DNA damage marker. We observed high γH2AX levels in USP32 knockout than other DUBs (Figure 1D). A prominent level of γH2AX foci formation was observed in most of the putative DUBs than mock (Figure 1E), while USP32-depleted cells showed more γH2AX foci than other DUBs (Figure 1E, panel 7).

### DUB-knockout library identifies USP32 as a protein destabilizer of SLC35F2

**Figure 2:**
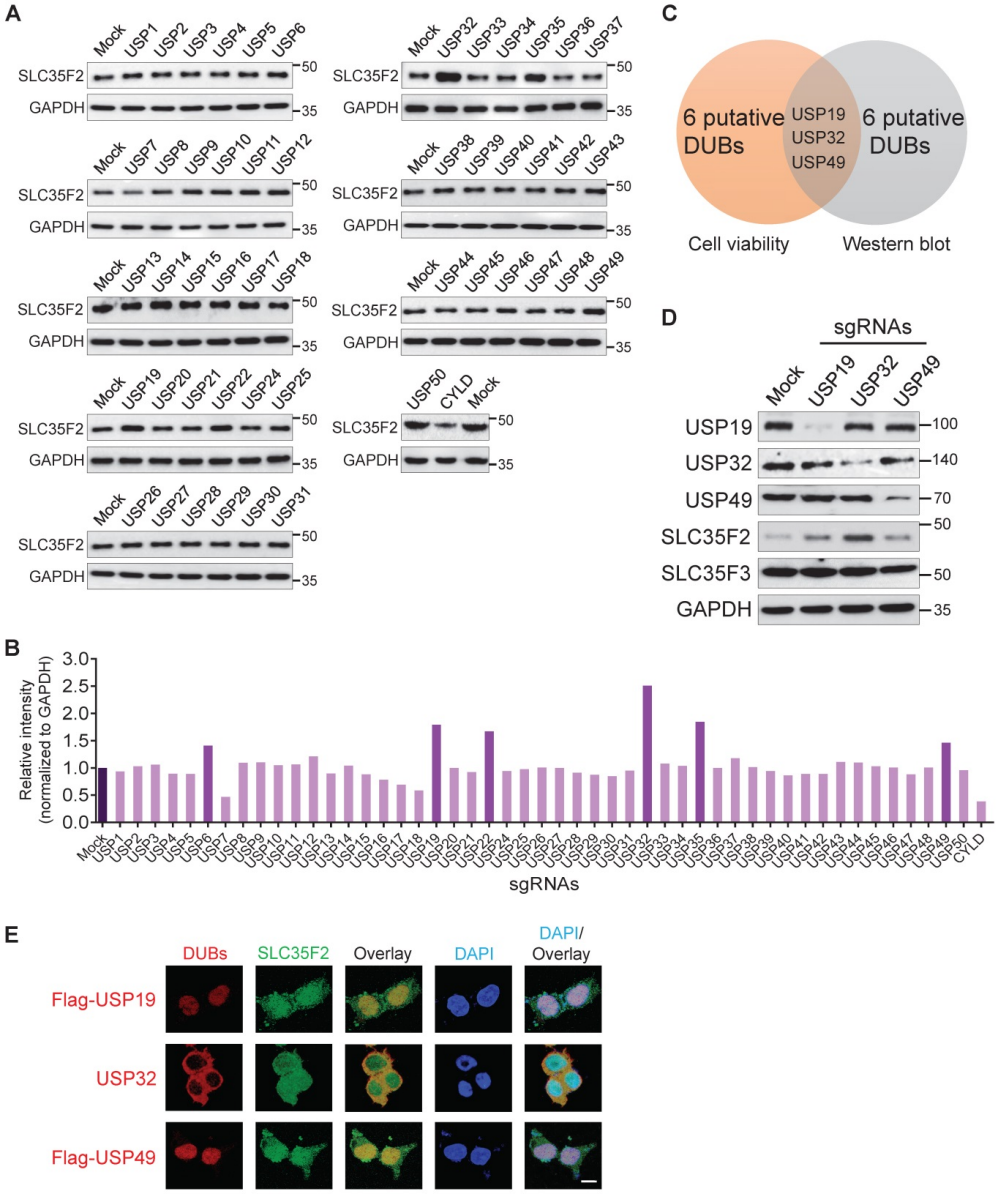
** DUB knockout library kit-based screening for USPs altering SLC35F2 protein stability through Western blot analysis. (A)** HeLa cells transfected with sgRNAs individually targeting entire set of genes encoding USPs along with Cas9 were subjected to western blot analysis to determine endogenous SLC35F2 protein levels. HeLa cells co-transfected with Cas9 and scrambled sgRNA served as mock controls. GAPDH was used as a loading control. **(B)** Relative protein expression of SLC35F2 was quantified using ImageJ software and represented as a bar graph. **(C)** A Venn diagram showing the overlapping candidates from our dual-screening approach (cell viability assay and western blot analysis). **(D)** Overlapping candidates were validated by western blot analysis. The sgRNAs targeting USP19, USP32, and USP49 were transfected in HeLa cells and a western blot was performed with the indicated antibodies. **(E)** An immunofluorescence staining assay was performed to analyze the co-localization behaviors of USP19, USP32, and USP49 with respect to SLC35F2 in HeLa cells. Because specific antibodies against USP19, USP49, and SLC35F2 are derived from the same species, we transfected HeLa cells with either Flag-USP19 or Flag-USP49 and then performed staining with an anti-Flag and SLC35F2-specific antibodies. Scale bar = 25 µm.

Previously, haploid genetic screening identified SLC35F2 as a YM155 drug-importer generating DNA damage in cancer cells 14. The differential expression patterns of SLC35F2 in several cancer cells induce YM155-mediated DNA damage is poorly understood. Here, we observed that knockout of several DUBs induces YM155-mediated DNA damage in cancer cells resistant to YM155 (Figure 1B-E). Thus, we performed the genome-scale DUB screening that regulate SLC35F2 expression. The DUB genes for which loss-of-function confers a reduction or increase in SLC35F2 protein were screened by analyzing SLC35F2 expression. We observed that sgRNAs targeting USP6, USP19, USP22, USP32, USP35, and USP49 were associated with increase in SLC35F2 protein than mock (Figure 2A) and presented graphically for clarity (Figure 2B).

Due to the fact that the increase in SLC35F2 protein directly affects YM155 uptake, we were interested in the putative DUBs upon knockout that resulted in increased SLC35F2 protein, as part of our effort to identify new cancer targets. Among DUBs, USP32 knockout significantly increased SLC35F2 protein (Figure 2A-B). Three candidates (USP19, USP32, and USP49) overlapped in both cell viability and western blot analysis (Figure 2C). Consistent with the above results, the negative regulation exhibited by USP19, USP32, and USP49 was highly specific to SLC35F2 but failed to stabilize its immediate family member, SLC35F3 (Figure 2D).

Co-localized gene pairs reportedly tend to have more interactions and regulate their functions 26. Thus, we examined the co-localization of USP19, USP32, and USP49 with respect to SLC35F2. We found that USP32 and SLC35F2 were predominantly co-localized in the plasma membrane, and some fraction of SLC35F2 was also localized in the nucleus (Figure 2E, middle panel). The sgRNA targeting USP6, USP22, and USP35 showed some increase in SLC35F2 expression (Figure 2A) but could not sensitize to YM155-mediated cytotoxicity (Figure 1B). This might be due to the localization patterns of USP6, USP22, and USP35 in the nucleus 27-29. Thus, membrane-localized behavior of USP32 is crucial for regulation of SLC35F2-mediated YM155 import activity. Altogether, our CRISPR-Cas9-based dual-screening strategies (cell viability and immunoblot analysis) identified USP32 as a dominant genetic determinant of YM155 drug resistance.

### USP32 negatively regulates SLC35F2 protein stability

**Figure 3:**
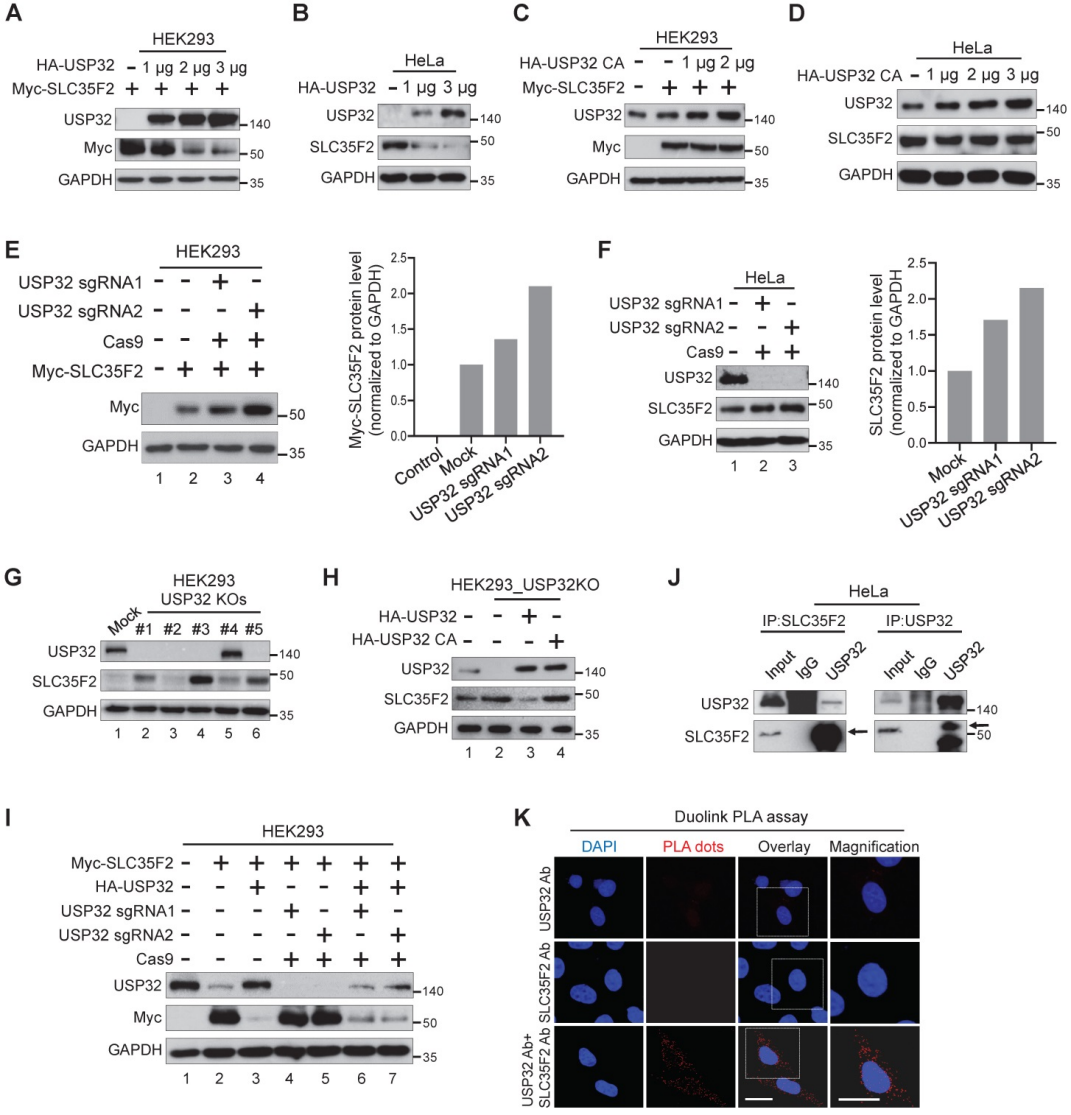
** USP32 impairs SLC35F2 protein stability. (A)** Exogenous protein levels of SLC35F2 in HEK293 cells were analyzed upon transfection with increasing concentrations of HA-USP32 along with Myc-SLC35F2. Western blot was performed to determine exogenous SLC35F2 expression. **(B)** The effect of USP32 on endogenous SLC35F2 protein expression was determined in HeLa cells transfected with increasing concentrations of HA-USP32. Western blot analysis was performed to determine endogenous SLC35F2 protein. **(C)** HEK293 cells transfected with increasing concentrations of USP32 C743A, along with Myc-SLC35F2 to check the effect of the catalytic mutant of USP32 (USP32 C743A). Western blot analysis was performed with the indicated antibodies. **(D)** The effect of USP32 C743A on endogenous SLC35F2 protein was analyzed upon transfection with increasing concentrations of USP32 in HeLa cells. Western blot analysis was performed with SLC35F2-specific antibodies. **(E)** HEK293 cells were co-transfected with Myc-SLC35F2 alone or in combination with USP32 sgRNA1 or USP32 sgRNA2. Western blot analysis was used to determine the exogenous SLC35F2 protein level. The graph in the right panel represents expression of the Myc-SLC35F2 protein. **(F)** Endogenous SLC35F2 protein levels were measured in the presence of sgRNAs targeting USP32 by western blot analysis in HeLa cells. The graph in the right panel represents endogenous expression of SLC35F2 protein. **(G)** Validation of single cell-derived USP32 knockout clones in HEK293 cells by western blot analysis. Cell lysates were collected and immunoblotted with respective antibodies to analyze endogenous expression of USP32 and SLC35F2. **(H)** HEK293_USP32KO cells were transfected with either HA-USP32 or HA-USP32 C743A plasmids to measure the reconstitution effect of USP32 on endogenous SLC35F2 protein by western blot analysis. **(I)** HEK293 cells were co-transfected with Myc-SLC35F2 alone or in combination with HA-USP32 or in the presence of sgRNAs targeting USP32 to check the reconstitution effect of USP32 on exogenous SLC35F2 expression by western blot analysis. **(J)** Endogenous interaction between USP32 and SLC35F2 was analyzed in HeLa cells. Cell lysates from HeLa cells were immunoprecipitated with USP32- or SLC35F2-specific antibodies and immunoblotted with indicated antibodies. **(K)** HeLa cells were subjected to Duolink PLA assay and stained with mentioned antibodies. Scale bar = 25 µm.

To determine whether USP32 destabilizes SLC35F2, we transfected HA-USP32 in a dose-dependent manner. We found that SLC35F2 was reduced in a dose-dependent manner at either exogenous (Figure 3A) or endogenous levels (Figure 3B). In contrast, dose-dependent increase of catalytic mutant USP32 (HA-USP32 C743A) had no impact on SLC35F2 levels at either exogenous (Figure 3C) or endogenous levels (Figure 3D).

We transfected USP32 sgRNAs to validate the knockdown effect of USP32 on SLC35F2 protein. SLC35F2 was upregulated when USP32 was depleted by sgRNA2 at either exogenous (Figure 3E, lane 4) or endogenous levels (Figure 3F, lane 3). We previously generated several stable USP32 knockouts in HEK293 cells using sgRNA2 22 and validated its knockout efficiency using western blot (Figure 3G). SLC35F2 was upregulated in most of the USP32 knockouts clones (Figure 3G). High SLC35F2 level was observed in clone #3 (hereafter HEK293_USP32KO) than mock (Figure 3G, lane 4) and used for further experiments. The overexpression of wild-type USP32 (Figure 3H, lane 3) but not catalytic mutant (Figure 3H, lane 4) reverses the SLC35F2 protein stabilization in HEK293_USP32KO. Similarly, we reconstituted USP32 in USP32-depleted cells and analyzed the Myc-SLC35F2 levels in HEK293 cells. USP32 overexpression destabilized SLC35F2 protein than mock (Figure 3I, lane 3). The sgRNAs targeting USP32 upregulated SLC35F2 protein (Figure 3I, lanes 4 and 5) and reconstitution reversed SLC35F2 stabilization (Figure 3I, lanes 6 and 7). We also examined whether USP32 interacts with SLC35F2 by immunoprecipitation with USP32 or SLC35F2 antibodies. The results showed that USP32 co-precipitated with SLC35F2 and vice versa (Figure 3J). Additionally, we demonstrated that USP32 and SLC35F2 interact with each other by Duolink PLA assay. As shown in Figure 3K, the *in situ* USP32-SLC35F2 interaction (PLA dots) was observed when USP32 and SLC35F2 were immunostained together but not when they were stained with USP32 or SLC35F2 antibody alone. These data suggest that USP32 interacts with SLC35F2 and negatively regulates its protein stability.

### SLC35F2 undergoes proteasome-mediated protein degradation

**Figure 4:**
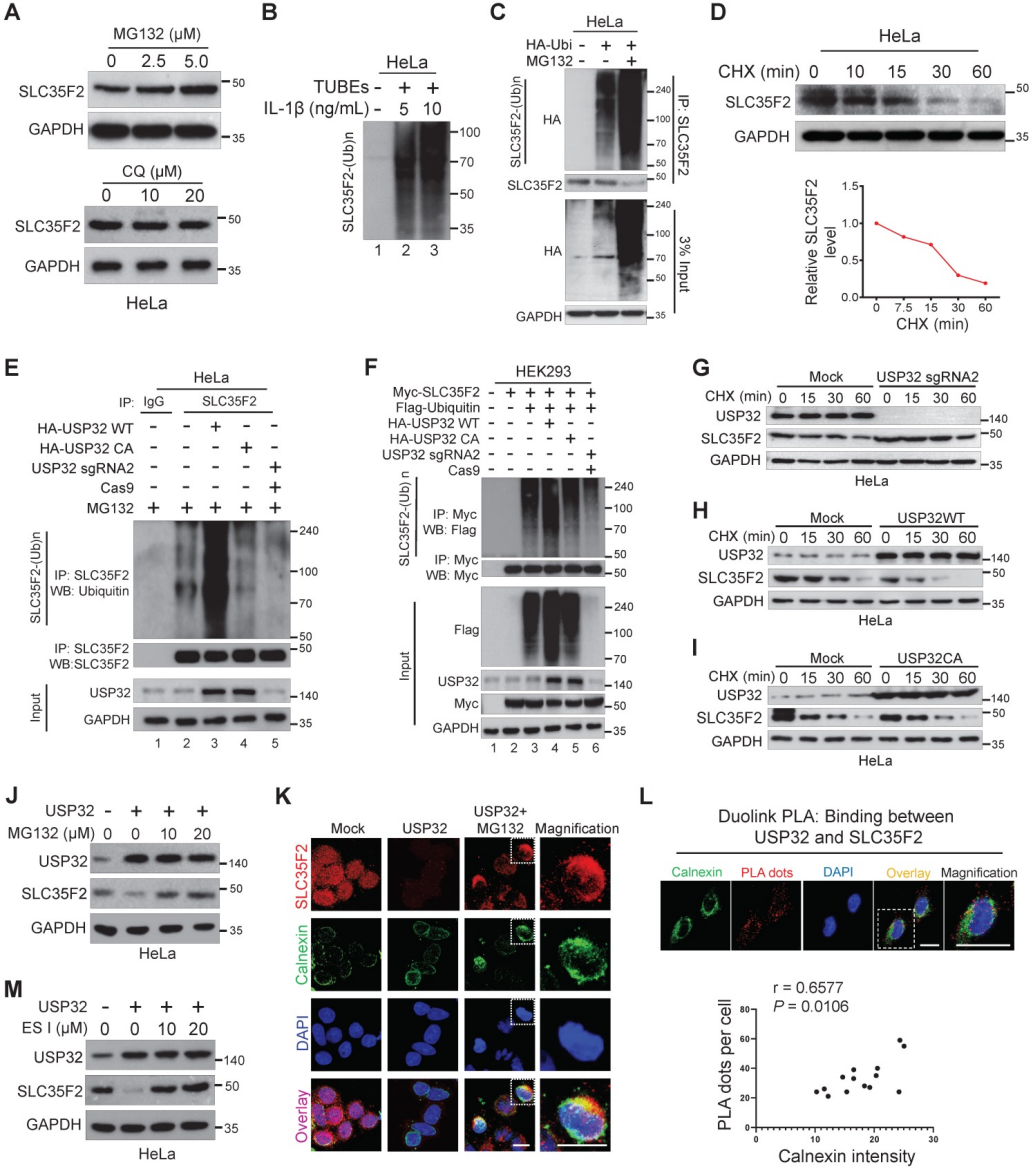
** SLC35F2 undergoes ubiquitination and USP32 promotes its ER-associated degradation. (A)** HeLa cells were treated with the indicated concentrations of MG132 for 4 h or with indicated concentrations of lysosomal inhibitor CQ. Western blot analyses were performed with the indicated antibodies. **(B)** HeLa cells were stimulated with 5 or 10 ng/mL of IL-1β for 10 minutes and lysed to capture polyubiquitinated chains from the cell extracts using TUBEs. **(C)** Endogenous ubiquitination of SLC35F2 was analyzed by transfecting HeLa cells with HA-ubiquitin. Immunoprecipitated with SLC35F2 antibody followed by immunoblotting with HA to monitor the ubiquitination status of endogenous SLC35F2. **(D)** HeLa cells were treated with 150 µg/mL of cycloheximide (CHX) for the indicated time points and harvested for western blot analysis with SLC35F2 antibodies. The rate of SLC35F2 protein decay was quantified and graphically represented using ImageJ (bottom panel). **(E)** HeLa cells transfected with indicated constructs and treated with 5 µM MG132 for 5 h before harvesting. Next, SLC35F2-specific antibodies were used to immunoprecipitated the protein, and specific ubiquitin antibodies were used for immunoblotting to check the polyubiquitination status of endogenous SLC35F2 in the presence and absence of HA-USP32 or HA-USP32 C743A. **(F)** HEK293 cells were transfected with the indicated constructs. Cells were then lysed and immunoprecipitated with Myc antibody and immunoblotted with Flag antibody. **(G-I)** HeLa cells were transfected with indicated constructs and incubated for 48 h. Next, 150 µg/mL of CHX was treated for the indicated time intervals and harvested for western blot analysis with the indicated antibodies. The rate of SLC35F2 protein decay was quantified in the presence of USP32 sgRNA2 or USP32 WT or USP32 CA and represented graphically using ImageJ (bottom panel). **(J)** Western blot analysis of HeLa cells transfected with USP32 and treated with or without MG132. **(K)** HeLa cells transfected with USP32 and treated with or without MG132 were immunostained with SLC35F2 antibody (Red), calnexin (Green), and DAPI (Blue). Scale bar = 10 µm. **(L)** HeLa cells were subjected to immunofluorescence analysis using calnexin (ER marker) antibody following Duolink PLA assay to check the location of USP32 interaction with SLC35F2. Scale bar = 25 µm. Bottom panel shows the correlation between USP32-SLC35F2 PLA dots and calnexin intensity derived from the Duolink PLA assay (n = 14). **(M)** Western blot analysis of HeLa cells transfected with USP32 and treated with or without ES I.

To investigate the catabolic activity of SLC35F2 membrane protein, we analyzed SLC35F2 expression in the presence of the proteasomal inhibitor MG132 or lysosomal inhibitor Chloroquine (CQ). MG132 dose-dependently increased SLC35F2 protein level whereas CQ failed to do so (Figure 4A). These results indicated that the protein level of SLC35F2 was regulated by proteasome degradation pathway. To study whether SLC35F2 undergoes ubiquitination, we carried out a TUBE assay, which has a high affinity toward ubiquitinated proteins 30. When stimulated with IL-1β in HeLa cells, SLC35F2 underwent ubiquitination than non-stimulated cells (Figure 4B, lanes 2-3). To further validate the SLC35F2 ubiquitination, we transfected HA-ubiquitin and performed IP with SLC35F2 antibody followed by immunoblotting using HA antibody (Figure 4C). SLC35F2 interacts with ubiquitin molecules and readily undergoes polyubiquitination and a similar result was observed at an exogenous level (Figure S1A, lane 3). The RT-qPCR analysis upon MG132 treatment in HeLa cells to examine SLC35F2 mRNA level showed no significant changes at the transcriptional level of SLC35F2 (Figure S1B), indicating SLC35F2 undergoes proteasome-mediated post-translational regulation.

Next, we monitored the decay kinetics of SLC35F2 protein using the protein synthesis inhibitor cycloheximide (CHX). SLC35F2 protein was degraded by CHX and its half-life was between 15 and 20 min. This is the first report explains SLC35F2 ubiquitination with a minimal half-life (Figure 4D).

### USP32 negatively regulates SLC35F2 abundance through the ERAD pathway and promotes polyubiquitination of SLC35F2

To examine the antagonistic nature of USP32 on SLC35F2 protein level, we performed an endogenous ubiquitination assay in the presence of wild-type or mutant USP32. Wild-type USP32 promoted SLC35F2 ubiquitination (Figure 4E, lane 3) but not catalytic mutant USP32 (Figure 4E, lane 4). Likewise, transient knockdown of USP32 diminished SLC35F2 ubiquitination (Figure 4E, lane 5). Similar results were also noticed at an exogenous level (Figure 4F). Thus, we hypothesized that USP32 may have an influence on the SLC35F2 protein turnover rate. We therefore transfected sgRNA targeting USP32 (USP32 sgRNA2), wild-type USP32 (USP32 WT), or mutant USP32 (USP32 CA) in HeLa cells and examined the decay kinetics of SLC35F2 protein. We found that the half-life of SLC35F2 protein in USP32-deficient cells was longer than mock (Figure 4G and Figure S1C), indicating that USP32 depletion extends the half-life of SLC35F2. In contrast, USP32 WT caused a severe reduction in the half-life of SLC35F2 when compared with mock (Figure 4H and Figure S1D) but mutant USP32 failed to modify SLC35F2 protein half-life (Figure 4I and Figure S1E).

In eukaryotes, the membranes proteins are mainly degraded by three distinct mechanisms. Endosomal sorting complexes required for transport and endosome/Golgi-associated degradation through lysosome, and endoplasmic reticulum (ER)-associated degradation (ERAD) through proteasome 31. To discover the cellular location where USP32 promoted SLC35F2 degradation, we transfected USP32 and treated with MG132 and found that inhibition of SLC35F2 stability by USP32 was blocked by MG132 (Figure 4J). SLC35F2 abundance remained unchanged when cells treated with CQ (Figure S1F), indicating that USP32 destabilizes SLC35F2 protein in the early stage of protein synthesis through the proteasome degradation pathway. By immunofluorescence, we also noticed that the restored SLC35F2 expression after MG132 treatment accumulated in the cytoplasmic region, which is co-localized with the ER membrane protein calnexin (Figure 4K). To further validate these findings, we performed Duolink PLA assay followed by immunofluorescence analysis using antibodies against the ER marker calnexin to explore the subcellular interaction of USP32 and SLC35F2. As shown in Figure 4L, the association of USP32 with SLC35F2 was co-localized by giving red dot signals along with the ER marker calnexin. The number of USP32-SLC35F2 PLA dots positively correlated with the fluorescence intensity of calnexin (Figure 4L, bottom panel). Consistently, addition of eeyarestatin I (ES I), a specific inhibitor of ERAD 32, to USP32 transfected cells significantly replenished SLC35F2 protein level (Figure 4M), suggesting that USP32 regulates SLC35F2 in the ER.

### Expression pattern of USP32 and SLC35F2 exhibits a negative correlation across a wide panel of cancer cells

**Figure 5:**
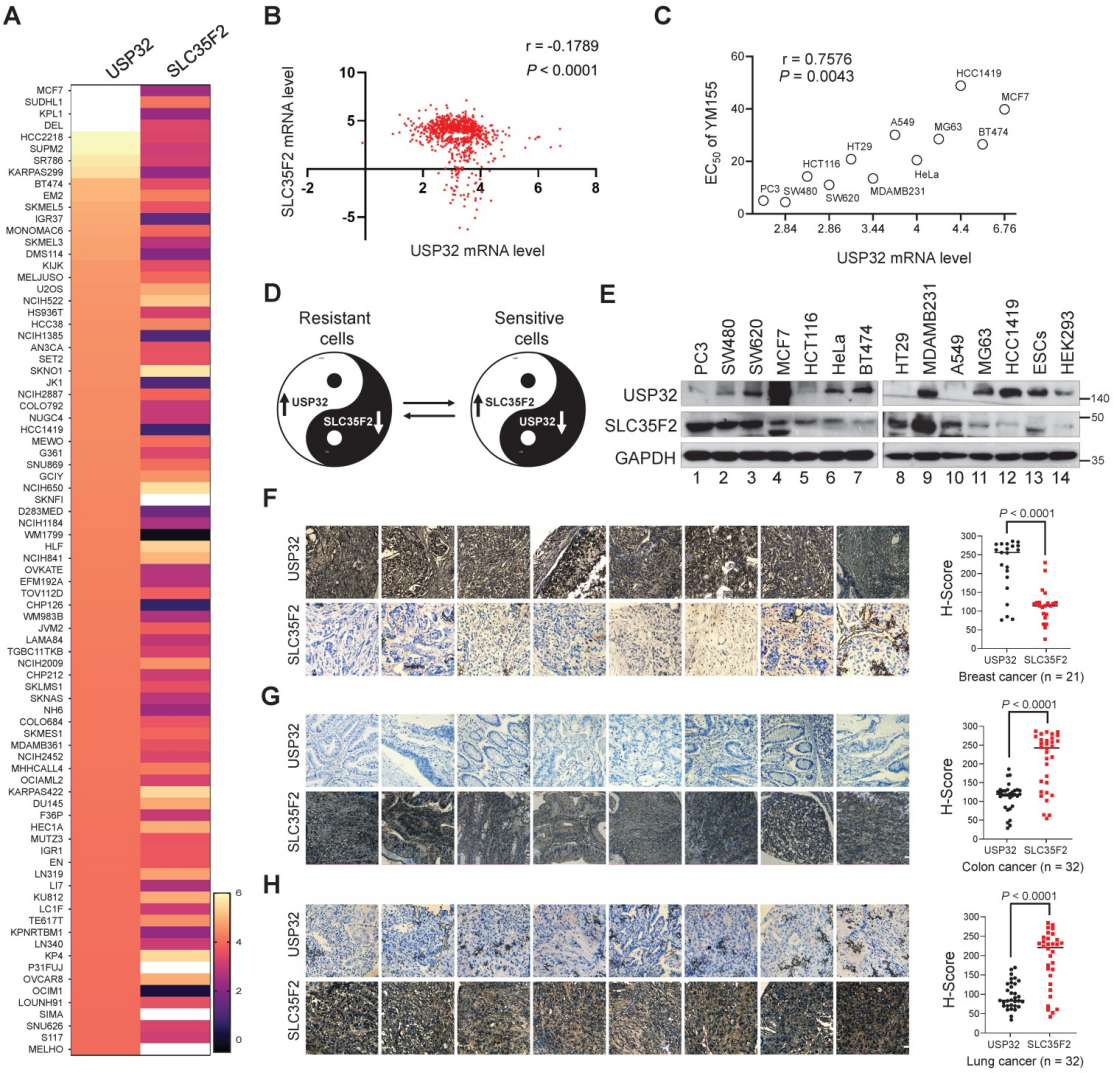
** USP32 and SLC35F2 expression patterns are negatively correlated across wide panel of cancer cells. (A)** A heat map showing mRNA expression levels of USP32 and SLC35F2 derived from the CCLE panel. Representative samples are arranged from high to low mRNA levels of USP32 and corresponding SLC35F2 values are sorted. **(B)** A scatterplot was made between USP32 and SLC35F2 mRNA levels. Pearson correlation analysis was used to quantify the relationship between USP32 and SLC35F2. **(C)** EC_50_ values for YM155 were derived for the indicated cell lines and presented as a function of USP32 mRNA expression levels. **(D)** A Yin-Yang model showing the resistant and sensitive patterns of cancer cells to YM155 drug. **(E)** Endogenous protein expression patterns of USP32 and SLC35F2 in different cancer and non-cancer cell lines were analyzed by western blot analysis. **(F-H)** Representative immunohistochemical (IHC) staining images of endogenous USP32 and SLC35F2 in human breast cancer (n = 21), colon cancer (n = 32) and lung cancer (n = 32) tissues. All IHC images were quantified by an *H* score. A two-tailed *t* test was used and *P* values are as indicated. Scale bar = 50 µm.

We analyzed the correlation between USP32 and SLC35F2 using the CCLE database. The high score for USP32 mRNA level in any given cancer cell line was inversely proportional to the SLC35F2 mRNA, which suggests a significant negative correlation between USP32 and SLC35F2 (Figure 5A) with a r value of -0.1789 (Figure 5B). However, there was no correlation between the high score for SLC35F2 mRNA level and USP32 mRNA (Figure S2A-B), suggesting that USP32 is a key factor for drug resistance in cancer cells. Given that USP32 is overexpressed in cancer cells, we speculated that the efficacy of YM155 uptake could be predicted by USP32 expression. First, we treated cancer cells with YM155 and derived half-maximal effective concentration (EC_50_) values. We then plotted USP32 mRNA expression profiles versus EC_50_ values for YM155 derived from the cell lines (Figure 5C). By comparing YM155 EC_50_ values to USP32 mRNA expression, we found a significant positive correlation over the tested cell lines (Figure 5C). This led us to hypothesize that the high USP32 expression and corresponding low SLC35F2 expression makes cancer cells resistant to YM155 and vice versa in case of sensitive cells. A Yin-Yang model represents the resistant and sensitive patterns of cancer cells to YM155 (Figure 5D). To corroborate the negative correlation between USP32 and SLC35F2, we estimated the expression of these two proteins in several cancers. We found that USP32 was elevated in MCF7 and BT474 cells was associated with low expression of SLC35F2 (Figure 5E, lanes 4 and 7). In contrast, low USP32-expressing cancer cells such as PC3, SW480 and HCT116 was associated with relatively high SLC35F2 expression (Figure 5E, lanes 1, 2 and 5), suggesting that the USP32 is negatively correlated with SLC35F2 protein in several human cancer cell lines (Figure 5E).

To further confirm the correlation, we analyzed USP32 and SLC35F2 expression in different human tumor tissues. The expression patterns of USP32 and SLC35F2 in human breast (n = 21), colon (n = 32), and lung cancers (n = 32) obtained from ISU Abxis tissue microarray were subjected to immunohistochemistry staining. USP32 was highly upregulated in breast cancer while SLC35F2 were significantly lower in these tissues (Figure 5F and Figure S3A). However, SLC35F2 was highly upregulated in colon (Figure 5G and Figure S3B, lower panel) and lung cancer tissues (Figure 5H and Figure S3C, lower panel) and relative low levels of USP32 were observed in the respective tissues (Figure 5G-H and Figure S3B-C, upper panels). Altogether, USP32 and SLC35F2 are negatively correlated with each other, a relationship that may provide a predictive tool for YM155-mediated DNA damage.

### USP32-abundant cells show resistance to YM155-mediated DNA damage

**Figure 6:**
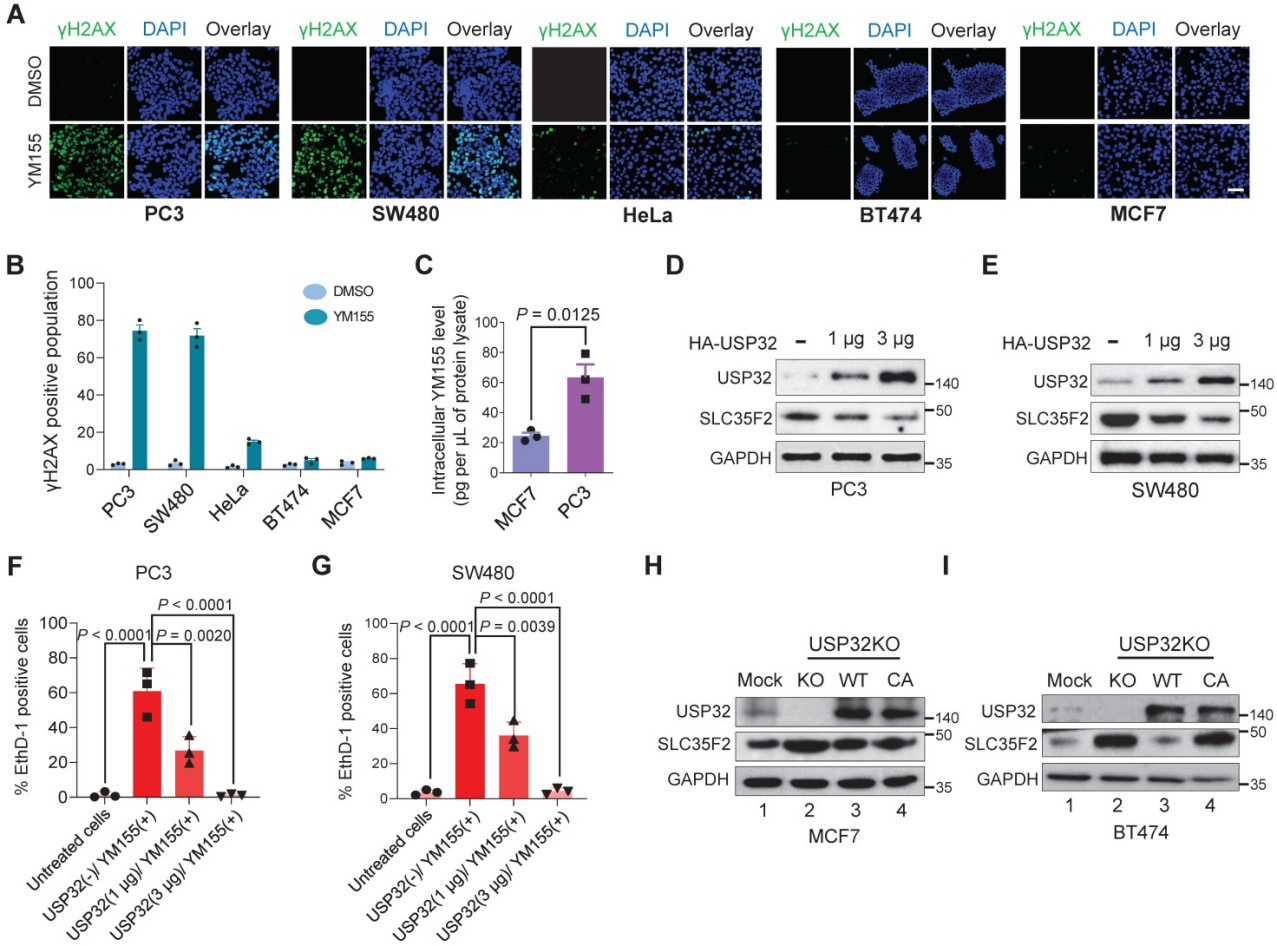
** USP32 expression confers resistance to YM155-mediated DNA damage. (A)** Immunofluorescence analysis of γH2AX foci formation in different cell lines treated with 25 nM YM155 for 24 h. **(B)** A quantification graph from (**A**) showing the percentage of the γH2AX-positive population in different cancer cell lines. Data are presented as the mean and standard deviation of three independent experiments. Scale bar = 50 µm **(C)** Intracellular uptake of YM155 drug levels was determined by multiple-reaction monitoring-mass spectroscopy analysis (MRM-MS) in MCF7 and PC3 cells exposed to 3 µM YM155 for 90 min. Data are presented as the mean and standard deviation of three independent experiments. A two-tailed *t* test was used and the *P* value is as indicated. **(D-E)** USP32 plasmids were dose-dependently transfected in PC3 (**D**) and SW480 (**D**) cell lines with high levels of endogenous expression of SLC35F2. Western blot analysis was performed with the indicated antibodies. GAPDH was used as a loading control. **(F-G)** Cells from (**D**) and (**E**) were subjected to YM155 treatment (25 nM) for 24 h and stained with EthD-1 to quantify the EthD-1-positive cells. Data are presented as the mean and standard deviation of three independent experiments. One-way ANOVA followed by Tukey's post hoc test was used and the *P* values are as indicated. **(H-I)** Validation of the single cell-derived USP32 knockout clones in MCF7 (**H**) and BT474 (**I**) cell lines using western blots with the indicated antibodies.

We confirmed the extent of DNA damage induced in different cell lines treated with YM155 by estimating the γH2AX levels. We selected five different cancer cell lines based on USP32 endogenous expression (PC3 < SW480 < HeLa < BT474 < MCF7) obtained from Figure 5E and treated with YM155. A pronounced increase in the γH2AX foci formation was detected in PC3 and SW480 cells, moderate γH2AX foci was formed in HeLa cells, and negligible γH2AX foci was formed in BT474 and MCF7 cells (Figure 6A). In line with immunoblot results (Figure 5E), reduced USP32 expression promoted YM155 sensitivity and thus γH2AX foci was formed.

Consistent with the above findings, USP32-overexpressing cells lines (MCF7 and BT474) were less potent after YM155 treatment, resulting in a decrease in the magnitude of the sub-G1 population, whereas SW480 and PC3 displayed a significant increase in the sub-G1 population (Figure 6B), suggesting reduced USP32 expression induces YM155-mediated cell apoptosis.

### USP32 negatively regulates SLC35F2-mediated cellular uptake of YM155 by limiting its protein abundance

To test the hypothesis that cellular uptake of YM155 is hindered by USP32 expression, we monitored intracellular drug uptake in cell lines expressing high (MCF7) and low (PC3) levels of USP32 using MRM mass spectroscopy (MS). After treatment with 3 µM YM155 for 120 min, we observed significantly lower levels of YM155 taken up by MCF7 compared with PC3 (Figure 6C). This may be attributable to the high endogenous expression of USP32 in MCF7 cells, resulting in SLC35F2 protein destabilization that causes low intracellular YM155 uptake.

Next, we checked whether USP32 is indeed encumbering YM155 cellular uptake by destabilizing SLC35F2 expression. To this end, we overexpressed USP32 in a dose-dependent manner in PC3 and SW480 cells. We observed that SLC35F2 expression was gradually reduced when USP32 was increased dose-dependently in both the cell lines (Figure 6D-E). Cells from Figure 6D-E were analyzed for YM155-mediated cell apoptosis by treating them with 25 nM YM155 and stained with EthD-1 to quantify dead cells. The PC3 and SW480 cells having high SLC35F2 levels facilitated YM155 uptake and resulted in apoptosis (Figure 6F-G). Our results indicate that dose-dependent overexpression of USP32 destabilizes SLC35F2, reducing the YM155-mediated apoptotic effect.

### Generation of single cell-derived knockout clones of USP32 in MCF7 and BT474 cells

Our findings indicate that USP32 is a main cause of SLC35F2 destabilization and declines the YM155 uptake in MCF7 and BT474 cells. We therefore hypothesized that USP32 knockout in these cells would reverse YM155 resistance by upregulating SLC35F2. We generated *USP32* gene knockouts in MCF7 and BT474 cells using CRISPR-Cas9. We designed sgRNA1 and sgRNA2 targeting exon 3 of *USP32* (Figure S4A) 22. T7E1 assay showed that sgRNA2 is more efficient than sgRNA1 (Figure S4B) and it was used to generate knockout clones. The *USP32* gene disruption was screened by T7E1 assay and western blot (Figure S4C-F). The immunoblot revealed that clone #12, #13, #16, and #17 in MCF7 (Figure S4E) and clone #6, #7, and #9 in BT474 (Figure S4F) showed complete disruption of USP32, while SLC35F2 expression was upregulated in the knockout clones than wild-type. We selected MCF7 clone #16 (hereafter MCF7_USP32KO) and BT474 clone #6 (hereafter BT474_USP32KO) having out-of-frame mutations (Figure S4G-H) for further experiments. Off-target analysis of sgRNA2 targeting USP32 in MCF7_USP32KO and BT474_USP32KO showed no non-specific cleavages (Figure S4I).

### Loss of USP32 reverses YM155 resistivity and induces apoptosis by inhibiting PI3K/AKT/mTOR signaling pathway

**Figure 7:**
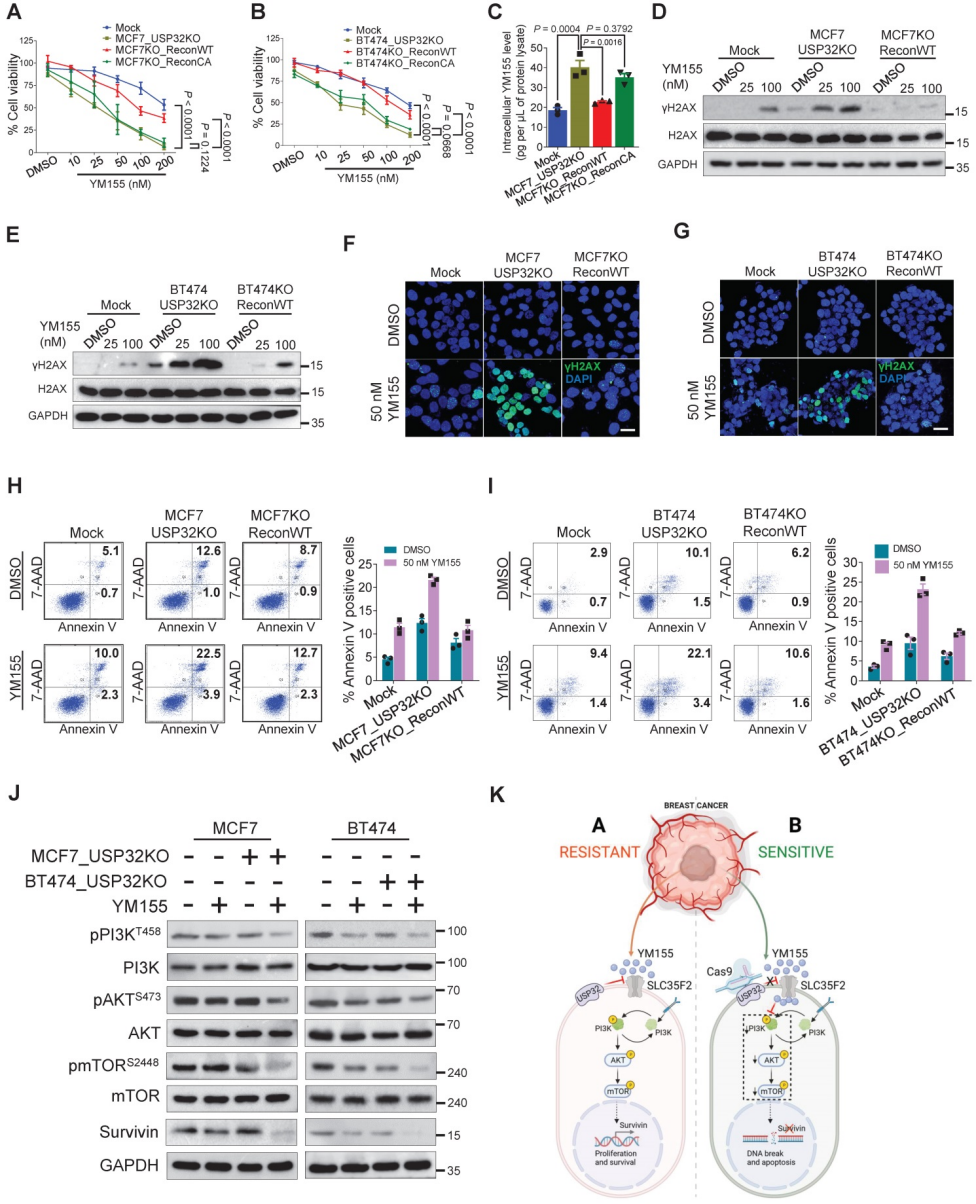
** Loss of USP32 enhances YM155-mediated DNA damage and apoptosis. (A)** The above MCF7-related cells were treated with the indicated concentrations of YM155 for 24 h and cell viability was measured. **(B)** The above BT474-related cells were treated with the indicated concentrations of YM155 for 24 h and cell viability was measured. **(C)** Intracellular uptake of YM155 drug levels were determined by MRM-MS in MCF7-related cells treated with 3 µM YM155 for 90 min. **(D)** MCF7-related cells were treated with either DMSO or indicated concentrations of YM155 for 24 h. **(E)** BT474-related cells were treated with either DMSO or indicated concentrations of YM155 for 24 h. Western blot analysis for (**D**) and (**E**) were performed with indicated antibodies. **(F)** MCF7-related cells were treated with either DMSO or 50 nM YM155 for 24 h and subjected to immunofluorescence analysis to estimate γH2AX foci formation. Green, γH2AX; blue, nucleus stained with DAPI. Scale bar = 25 µm. **(G)** BT474-related cells were treated with either DMSO or 50 nM YM155 for 24 h and subjected to immunofluorescence analysis to estimate γH2AX foci formation. Green, γH2AX; blue, nucleus stained with DAPI. Scale bar = 25 µm. **(H)** MCF7-related cells were treated with either DMSO or 50 nM YM155 for 24 h. **(I)** BT474-related cells were treated with either DMSO or 50 nM YM155 for 24 h. The cells from (**H**) and (**I**) were then stained with annexin V/7-AAD to monitor apoptotic populations by flow cytometry. **(J)** MCF7 and BT474-related cells were treated with or without 50 nM YM155 for 24 h and Western blot was performed with indicated antibodies. **(K)** Schematic representation showing the resistant and sensitive models of YM155-mediated cancer therapy. Data mentioned in (**A-C**, **F-I**) are presented as the mean and standard deviation of three independent experiments. One-way or Two-way ANOVA followed by Tukey's post hoc test was used and *P* values are as indicated.

We used USP32-knockout MCF7 and BT474 cells to demonstrate that loss of USP32 could increase SLC35F2 levels and mediate YM155 uptake. The USP32 knockout showing high SLC35F2 level were reconstituted with wild-type and mutant USP32 to measure the USP32 and SLC35F2 levels. Reconstitution of wild-type USP32 but not mutant USP32 destabilized SLC35F2 protein in USP32-knockout MCF7 (Figure 6H, lanes 3 and 4) and BT474 cells (Figure 6I, lanes 3 and 4). We also used the USP32-knockout cells to validate the USP32 dependence for YM155 uptake. MCF7_USP32KO and BT474_USP32KO cells decreased cell viability significantly than mock (Figure 7A-B). However, reconstitution of wild-type USP32 in MCF7_USP32KO and BT474_USP32KO cell lines (hereafter MCF7KO_ReconWT and BT474KO_ReconWT, respectively) decreased the YM155 uptake that leads to less sensitivity. Reconstitution with the USP32 mutant (hereafter MCF7KO_ReconCA and BT474KO_ReconCA, respectively) failed to alter the YM155 uptake than parent isogenic clones.

We assessed whether USP32 facilitates cellular uptake of YM155 by measuring intracellular drug concentrations using MRM-MS in MCF7 cells. When we treated 4 µM YM155 for 3 h, a significant increase in intracellular YM155 levels in MCF7_USP32KO cell line was observed (Figure 7C). However, MCF7KO_ReconWT cells not MCF7KO_ReconCA cells, exhibited decreased YM155 uptake than MCF7_USP32KO cells, suggesting that USP32 depletion enhances SLC35F2 level, which in turn improves YM155 uptake in MCF7 cells. Next, we validated the DNA damage by YM155 treatment in MCF7 and BT474 cells. A significant dose-dependent increment of γH2AX expression in MCF7_USP32KO and BT474_USP32KO cells was observed (Figure 7D-E). Likewise, the dose-dependent induction of DNA damage was impeded in MCF7KO_ReconWT and BT474KO_ReconWT cells than MCF7_USP32KO and BT474_USP32KO cells. Consistent with the above findings, γH2AX foci formation was increased in YM155-treated MCF7_USP32KO and BT474_USP32KO cells than mock (Figure 7F-G; middle panel). Reconstitution of wild-type USP32 in YM155-treated MCF7_USP32KO and BT474_USP32KO cells showed less γH2AX foci-positive cells (Figure 7F-G; right panel).

Next, we verified the ability of YM155 to induce apoptosis in USP32-depleted MCF7 and BT474 cells. The USP32 depletion resulted in increased sub-G1 populations (Figure S5A-B) and annexin-V-positive cells (Figure 7H-I) whereas it was considerably less toxic in corresponding wild-type cells. However, MCF7KO_ReconWT and BT474KO_ReconWT cells reverted to the apoptotic phenotype (Figure 7H-I; Figure S5A-B). Since the PI3K/AKT/mTOR axis is the fundamental for the regulation of cell proliferation and linked to multidrug resistance in cancer 33,34, we next investigated whether loss-of-USP32 can modulate this pathway. YM155 treated MCF7_USP32KO and BT474_USP32KO cells inhibited the activation of PI3K, AKT and mTOR as indicated by the reduction in pPI3K, pAKT and pmTOR expression (Figure 7J). Since YM155 originally was developed as a survivin suppressant 35, we assessed the expression of survivin in our experimental groups. As shown in Figure 7J, the protein level of survivin was significantly reduced in YM155-treated MCF7_USP32KO and BT474_USP32KO cells compared to YM155-treated MCF7 and BT474 cells. In summary, USP32 knockout sensitized breast cancer cells to YM155-mediated DNA damage and promoted cell apoptosis by inhibiting the PI3K/AKT/mTOR pathway and survivin protein level (Figure 7K).

### Loss of USP32 inhibits tumor progression *in vitro* and *in vivo* by promoting cellular uptake of YM155

**Figure 8:**
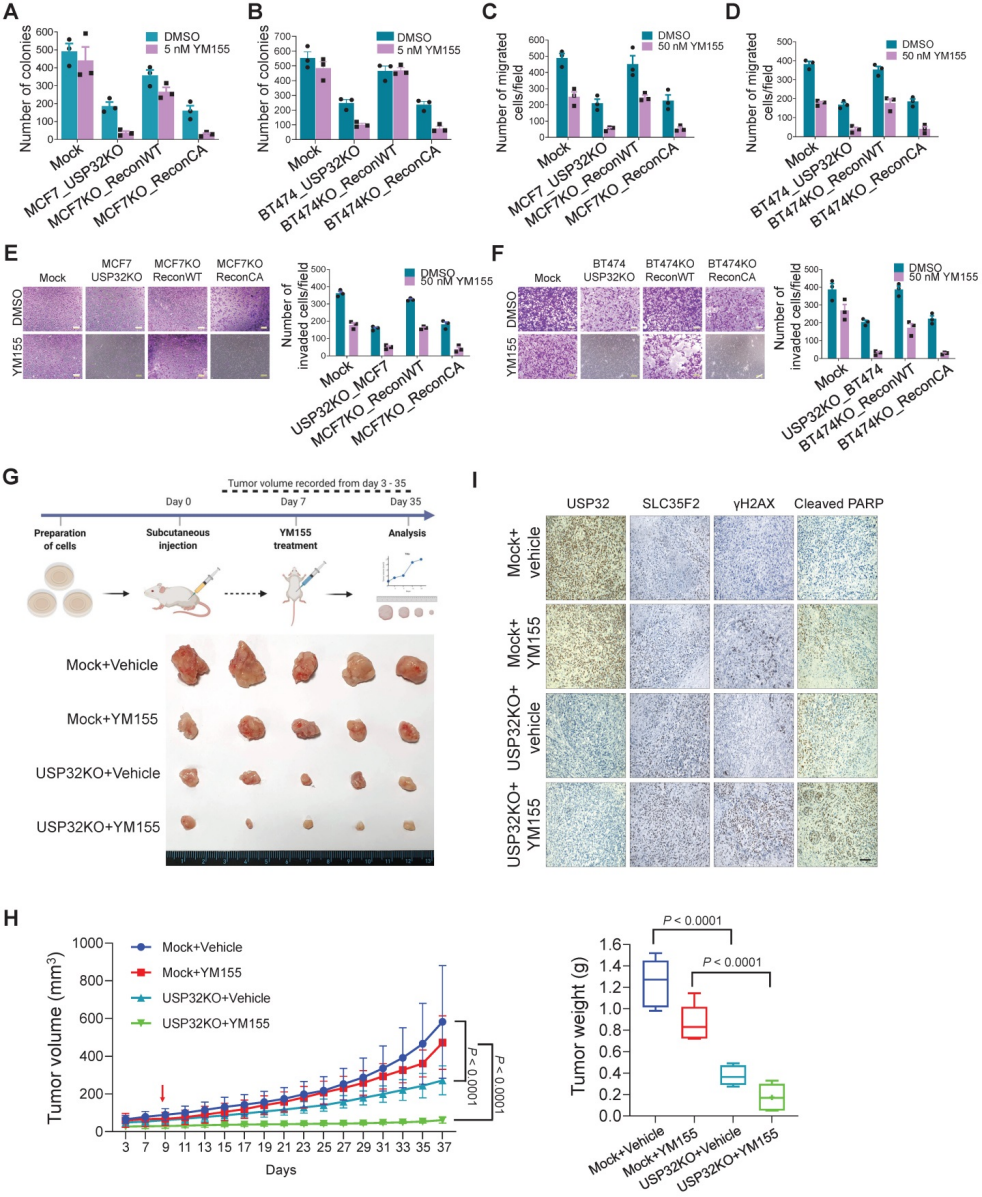
** USP32 knockout inhibits tumor progression *in vitro* and *in vivo*.** Colony formation assay was performed for **(A)** MCF7 mock, MCF7_USP32KO, MCF7KO_ReconWT, and MCF7KO_ReconCA cells; **(B)** BT474 mock, BT474_USP32KO, BT474KO_ReconWT, and BT474KO_ReconCA cells treated with either DMSO or 5 nM of YM155 for 14-days. The number of colonies was quantified and is presented graphically. Data are presented as the mean and standard deviation of three independent experiments. **(C-D)** Transwell cell migration assays were performed for the indicated cells treated with either DMSO or 50 nM YM155 for 24 h. Migrated cells were quantified using ImageJ. Data are presented as the mean and standard deviation of three independent experiments. **(E-F)** Transwell cell-invasion assays were performed for the indicated cells treated with either DMSO or 50 nM YM155 for 24 h. Invaded cells were quantified using ImageJ. Data are presented as the mean and standard deviation of three independent experiments. The number of invaded cells per field was quantified and represented graphically (right panels). **(G)** MCF7 wild-type and USP32 KO cell lines were prepared and subcutaneously injected into the right flank of NSG nude mice (n = 5). At day 7, mice were randomized into four groups as indicated and intraperitoneally injected with either saline (vehicle) or YM155 (7.5 mg/kg) twice in a week. Tumor volumes were recorded and stored for IHC experiments. The bottom panel shows the tumors excised from the mice after the experiment. **(H)** Tumor volume was measured every other day and is presented graphically. Data are presented as the mean and standard deviation (n = 5). Two-way ANOVA followed by Tukey's post hoc test was used and *P* values are as indicated. **(I)** Xenograft tumor tissues were sectioned and embedded in paraffin. Immunohistochemical analysis was performed with the indicated antibodies. Scale bar = 50 µm.

To test the hypothesis that USP32 governs YM155 efficacy *in vitro* and *in vivo*, we performed colony formation assay with long-term YM155 treatment (5 nM). We observed a decrease in colony numbers in MCF7_USP32KO and BT474_USP32KO cells treated with YM155 than MCF7 (Figure 8A; Figure S6A) and BT474 cells (Figure 8B; Figure S6B). Meanwhile, MCF7KO_ReconWT or BT474KO_ReconWT but not MCF7KO_ReconCA or BT474KO_ReconCA reduced YM155 toxicity, leading to an increased colony numbers (Figure 8A-B; Figure S6A-B). Likewise, cellular migration and invasion of YM155-treated USP32-depleted cells was significantly hampered. Reconstitution by wild-type USP32 in MCF7_USP32KO and BT474_USP32KO cells greatly improved migration (Figure 8C-D) and invasion (Figure 8E-F), although introduction of mutant USP32 failed to do so (Figure 8C-F).

We observed apoptosis in USP32-depleted cells, as evidenced by the accumulation of sub-G1 populations and annexin-V-positive cells (Figure 7H-I; Figure S5A-B). Additionally, a significant reduction in colony numbers, migration, and invasion was observed than mock controls (Figure 8A-F), suggesting that USP32 depletion itself can induce apoptosis.

Next, *in vivo* studies showed that YM155-treated USP32KO mice experienced significant reductions in tumor volume and weight than mock groups that received YM155 (Figure 8G-H; Figure S7). USP32KO mice groups that received saline alone showed reduced tumor formation than mock groups that received saline. USP32 expression was decreased in USP32KO groups and SLC35F2 expression was upregulated in these tumor tissues (Figure 8I). Selective induction of γH2AX expression in response to DNA damage after YM155 treatment was evaluated in these xenografts. USP32KO tumor group supplied with YM155 displayed more γH2AX expression and cleaved PARP than mock group supplied with YM155 (Figure 8I). Altogether, the depletion of USP32 enhances YM155-mediated sensitivity by stabilizing SLC35F2 protein, which hampers tumor progression.

## Discussion

Clinically evaluated anti-cancer drugs, of which YM155 is a notable entity, exhibit improved treatment outcomes in phase I and II clinical trials 7,36. The target induction of DNA damage by YM155 largely requires SLC35F2 expression to augment intracellular drug-import activity 14. Unfortunately, there are several cancers having low SLC35F2 levels, which hampers YM155 uptake and results in a low success rate in chemotherapeutics. To confront this problem, we screened for DUBs that can upregulate or stabilize SLC35F2 proteins in cancers exhibiting YM155 resistance due to low SLC35F2 expression.

Here, CRISPR-based dual-screening strategies (cell viability and protein stabilization) successfully identified USP32 as a novel and bona fide candidate that governs resistance for YM155 uptake by destabilizing SLC35F2 protein levels in a wide range of cancer cells. For the first time, we reveal that SLC35F2 undergoes ubiquitin-mediated proteasomal degradation and USP32 signals for rapid SLC35F2 degradation.

Previous studies have reported that USP32 is expressed at high level in several cancers, include breast cancer 37, small-cell lung cancer 38, and gastric carcinomas 39. In contrast, SLC35F2 is expressed highly in bladder 40, papillary thyroid 41, lung 42, and non-small-cell lung cancer 43. Based on these variable expression profiles exhibited by USP32 and SLC35F2, the ERAD-mediated proteasomal degradation of SLC35F2 was promoted by USP32. The main reason behind this regulation is the co-localization of USP32 and SLC35F2 on plasma membrane. For instance, in Western blot-based screening, we found that sgRNA targeting USP6, USP19, USP22, USP32, USP35, and USP49 increased the protein level of SLC35F2. However, USP6, USP22, and USP35 failed to increase the cytotoxicity of YM155. This might be due to the nuclear localization of USP6, USP22, and USP35 27-29,44. However, the increase in SLC35F2 protein level by USP6, USP22, and USP35 might have a different role in regulation of SLC35F2 protein function, which is an interesting topic for future studies. Thus, we performed our investigation based on membrane-localization between USP32 and SLC35F2 and its functional consequences in the regulation of YM155 cellular uptake.

The ubiquitin proteasome system (UPS) is one of the most important targets that impart cancer drug resistance 45,46. Thus, the UPS has been proposed as a potential target for new anti-cancer medicines, and small molecules are used to inhibit the proteolytic activities of this key cellular mechanism 47,48. Growing bodies of evidence demonstrate that the DUBs promote ubiquitination of their substrates only when they are interacting with each other 49,50. Thus, in this study, it was necessary to demonstrate the interaction between USP32 and SLC35F2 and its functional consequence on protein turnover of SLC35F2. We demonstrated by immunoprecipitation and immunofluorescence assays that USP32 destabilizes SLC35F2 protein level by interacting endogenously with it (Figure 3). The strong interaction between USP32 and SLC35F2 motivated us to analyze the effect of USP32 on protein ubiquitination and turnover of SLC35F2. The ubiquitination of SLC35F2 was significantly increased upon introduction of USP32 (Figure 4E-F). On the contrary, when USP32 was knocked down, the level of ubiquitination of SLC35F2 was inhibited drastically (Figure 4E-F). Moreover, we demonstrated that USP32 and SLC35F2 interact at the ER to promote SLC35F2 protein degradation via the ERAD pathway (Figure 4L). These results indicate that the interaction between USP32 and SLC35F2 promotes ubiquitination status of SLC35F2 protein.

USP32 is a poorly characterized DUB family member and was identified previously as a membrane-bound protease overexpressed in breast cancers 37. However, several studies have reported that overexpression of USP32 promotes tumorigenesis and drug resistance in different cancer types 38,39. Depletion of USP32 drastically reduced cell proliferation, migration, and invasion in the aforementioned cancers 38,39. Consistent with the results of previous studies, we found that knocking out USP32 alone in MCF7 and BT474 cells significantly reduced tumorigenic activity (Figure 8). MCF7 cells reportedly are less sensitive to YM155 51,52, and our correlation analysis between the EC_50_ of YM155 and USP32 mRNA levels revealed that MCF7 cells are less susceptible to YM155 toxicity due to overexpression of USP32 (Figure 5C). To test this hypothesis, we knocked out USP32 in both MCF7 and BT474 cells. Our results showed that depletion of USP32 in these cell lines induced DNA damage by enriching YM155 sensitivity both *in vitro* and *in vivo* (Figure 7 and 8).

Overall, our DUB-screening strategy revealed a resistance mechanism governed by USP32 associated with YM155 resistance in breast cancers, one that presents an attractive molecular target for anti-cancer therapies. Our USP32 knockout cell lines (MCF7_USP32KO and BT474_USP32KO) displayed sensitivity to YM155 and thereby caused survivin inhibition and DNA damage. Our approach to inhibiting USP32 to increase chemotherapeutic efficacy can be expanded to YM155-based cancer treatment in cells resistant to YM155 because of low SLC35F2. Applying our DUB-screening strategy to the identification of novel DUBs conferring resistance to several other potential small-molecule drugs scheduled for cancer therapy may increase its clinical efficacy, particularly during post-clinical stages. We envision that this approach will improve our understanding and ability to overcome drug resistance exhibited by several cancers and may open to avenues for chemotherapeutics.

## Supplementary Material

Supplementary figures.
