# Supplementary material for: USP32 confers cancer cell resistance to YM155 via promoting ER-associated degradation of solute carrier protein SLC35F2
Source: Theranostics. 2021 Sep 27;11(20):9752–71. doi: 10.7150/thno.63806 (PMC8581437; doi:10.7150/thno.63806)
Supplement: Supplementary file 1 — Supplementary figures. [file thnov11p9752s1.pdf]

USP32 promotes cancer cell resistance to YM155 via promoting ER-associated degradation of solute carrier protein SLC35F2

Arun Pandian Chandrasekaran[a], Kamini Kaushal[a], Chang-Hwan Park[a,b], Kye-Seong Kim[a,b,*], and Suresh Ramakrishna[a,b,*]

[a]Graduate School of Biomedical Science and Engineering, Hanyang University, Seoul, South Korea

[b]College of Medicine, Hanyang University, Seoul, South Korea

Running title: Depletion of USP32 enhances YM155-mediated DNA damage

*Corresponding authors

SR (E-mail: suri28@hanyang.ac.kr, suresh.ramakris@gmail.com);

Graduate School of Biomedical Science and Engineering, Department of Biomedical Science, Hanyang University, 222 Wangsimni-ro, Seongdong, Seoul 04763, Republic of Korea. Tel. +82 2 2220 2424, Fax. +82 2 2220 2422.

KS (E-mail: ks66kim@hanyang.ac.kr)

Graduate School of Biomedical Science and Engineering, Department of Biomedical Science, Hanyang University, 222 Wangsimni-ro, Seongdong, Seoul 04763, Republic of Korea. Tel. +82 2 2220 0601, Fax. +82 2 2220 2422.

Supplementary Figures

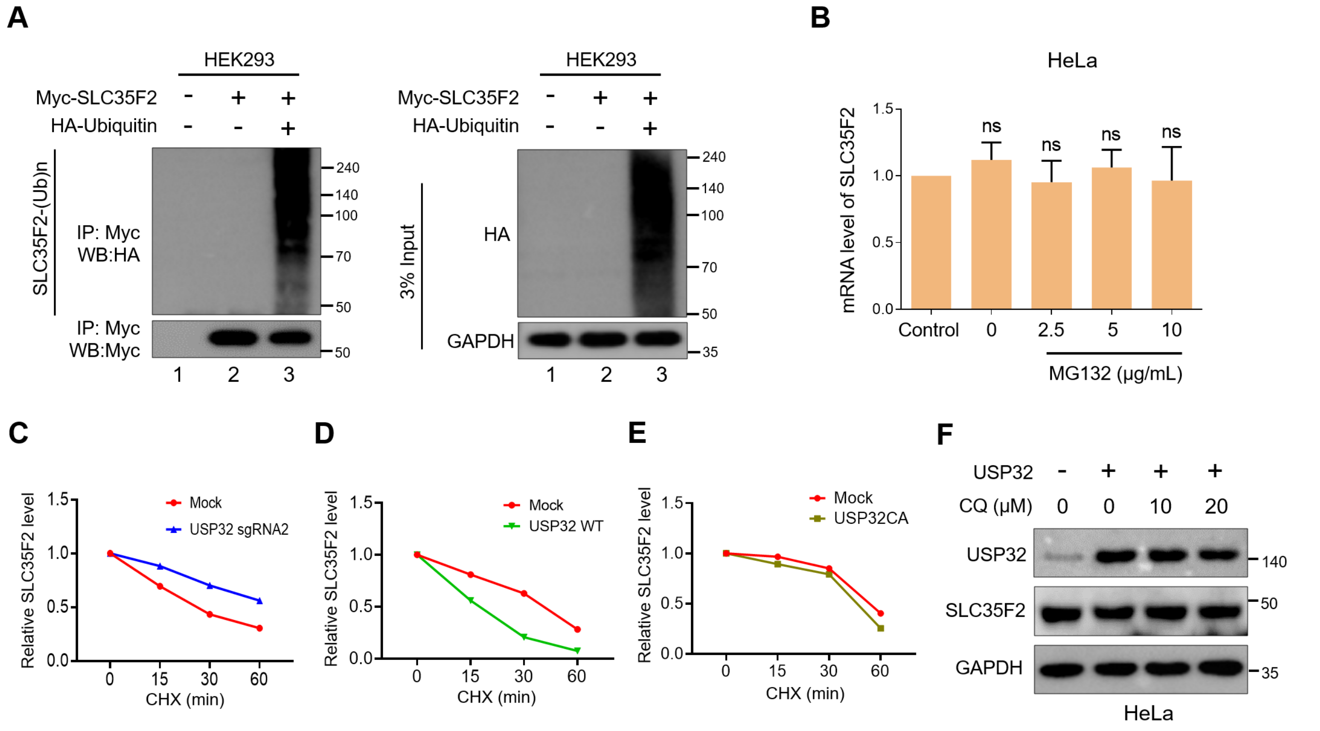

Figure S1. SLC35F2 undergoes 26S proteasome-mediated ubiquitination. (A) HEK293 cells were transfected with Myc-SLC35F2 alone or in combination with HA-ubiquitin. Immunoprecipitated with Myc antibody followed by immunoblotting with HA to assess the ubiquitination status of exogenous SLC35F2. **(B)** HeLa cells were treated with indicated concentrations of MG132 for 4 h. RT-qPCR was used to assess the mRNA expression levels of SLC35F2. Data are presented as the mean and standard deviation of three independent experiments. One-way ANOVA followed by Tukey's post hoc test was used and *P* values are as indicated. **(C-E)** Decay kinetics of relative SLC35F2 protein level in HeLa cells. **(F)** HeLa cells were treated with the indicated concentrations of CQ and Western blot analyses were performed with the indicated antibodies.

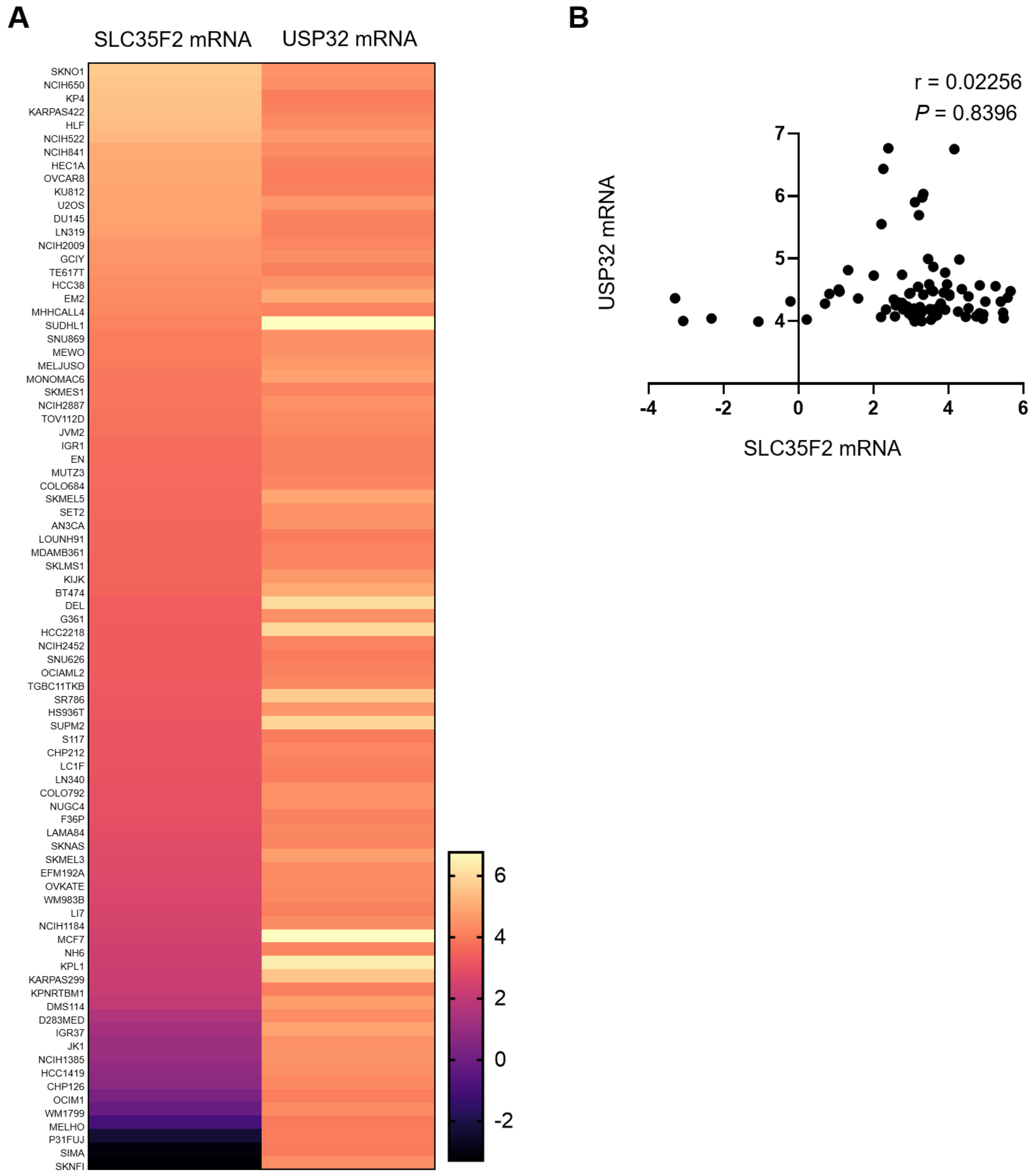

Figure S2. Correlation between SLC35F2 mRNA and USP32 mRNA levels. (A) A heat map showing mRNA expressions of SLC35F2 and USP32 derived from the CCLE panel. Representative samples are arranged from high to low mRNA levels of SLC35F2

and corresponding USP32 mRNA values are sorted. **(B)** A scatterplot was made between SLC35F2 and USP32 mRNA values. Pearson correlation analysis was used to quantify the relationship between SLC35F2 and USP32.

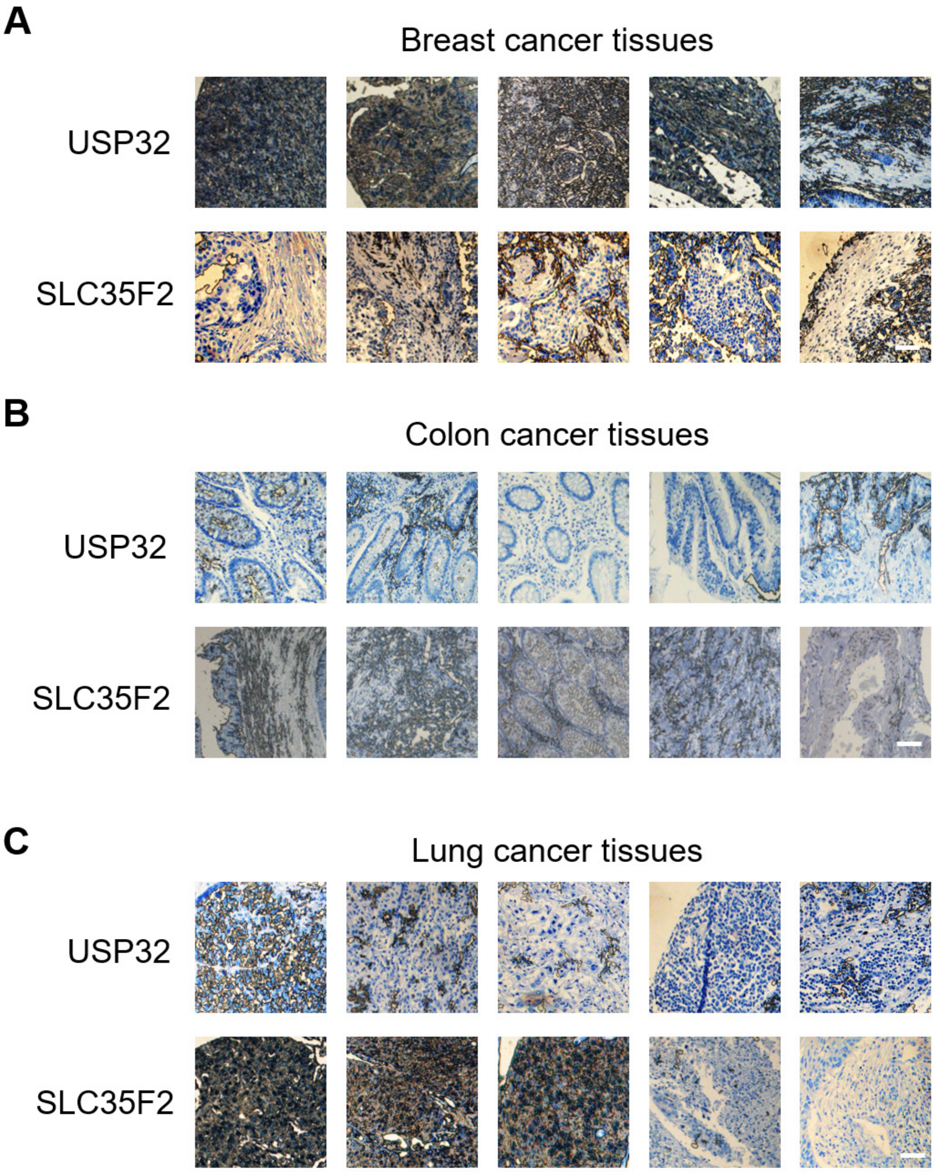

Figure S3: (A-C) Representative immunohistochemical staining images of endogenous USP32 and SLC35F2 expressions in human breast (**A**), colon (**B**) and lung (**C**) cancer tissues. Scale bar = 50 μm.

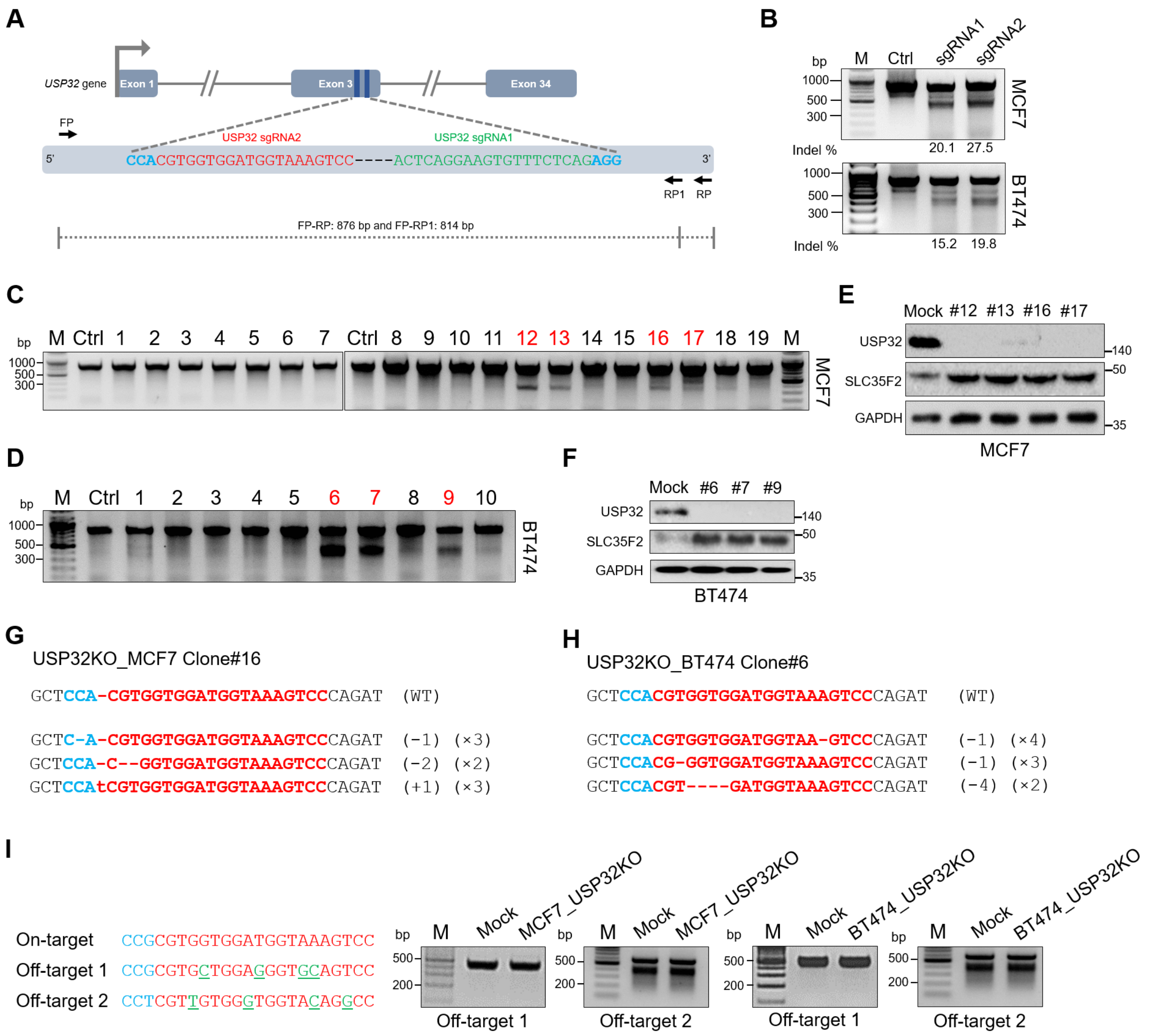

Figure S4. Generation of single cell–derived USP32 knockout clones in MCF7 and BT474. (A) Schematic representation of the sgRNA design strategy at exon 3 of *USP32* gene. sgRNA sequences are in green (sgRNA1) and red (sgRNA2); PAM sequences are in blue. The PCR amplicon is indicated by the dotted line with the expected size (814 bp). **(B)** Knockout efficiencies of the designed sgRNAs are validated in MCF7 and BT474 cells by T7E1 assay. Untransfected cells served as negative control (Ctrl), and the size marker (M) is shown. **(C-D)** T7E1 screening of stable knockout clones in MCF7

and BT474 cells. Untransfected cells served as negative control (Ctrl), and the size marker (M) is shown. Positive clones are indicated in red. **(E-F)** Endogenous protein expression of USP32 and SLC35F2 was validated in single cell–derived USP32 knockout clones from MCF7 and BT474 cells by western blot analysis. **(G-H)** *USP32* gene sequences from T7E1-positive clones in (**G**) MCF7 and (**H**) BT474 cells were confirmed by Sanger sequencing. The sgRNA recognition site and PAM sequences are in red and blue, respectively. The number of deleted bases is in the parentheses on the right. The number of occurrences is shown in parentheses (e.g. ×1, ×2, or ×3 indicate the number of each sequence). **(I)** Off-target analysis of USP32 knockout clones (MCF7_USP32KO and BT474_USP32KO). PAM sequences are shown in blue and mismatched bases are underlined in green. The mutation frequencies at off-target sites were determined using T7E1 assays.

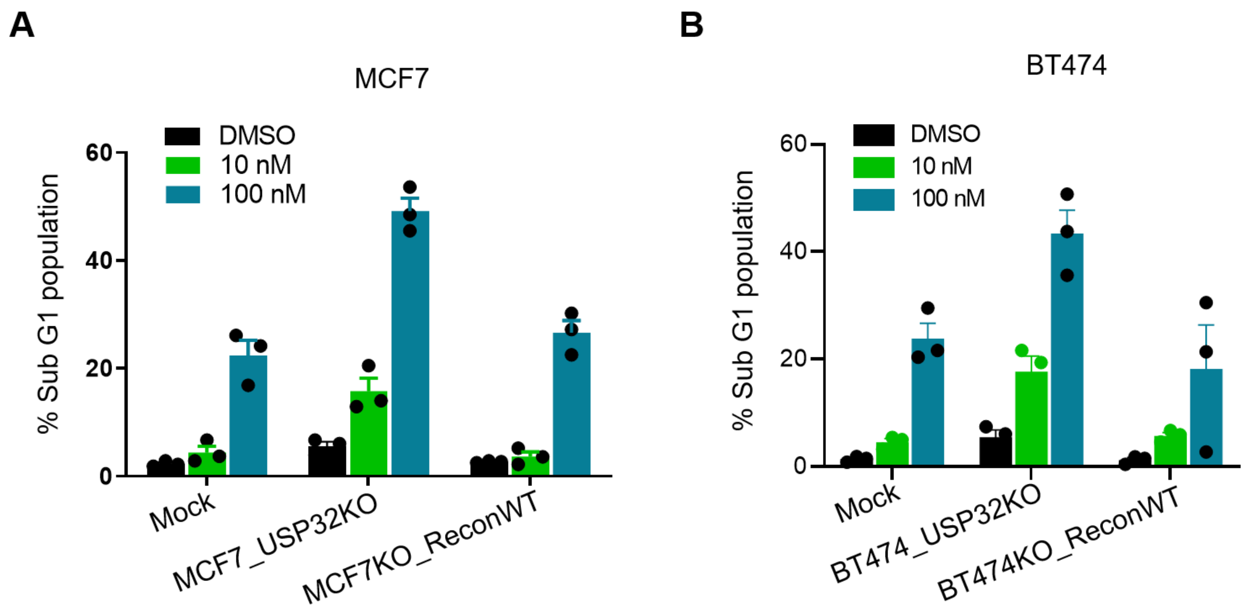

Figure S5. Apoptosis assays in MCF7 and BT474 cells. (A) DMSO or the indicated concentrations of YM155 was treated in wild-type MCF7 cells transfected with empty-vector (mock), MCF7_USP32KO, and MCF7KO_ReconWT cells for 24 h. The percentage of the sub-G1 population was measured by flow cytometry. Data are presented as mean and standard deviation of three independent experiments. **(B)** DMSO or the indicated concentrations of YM155 was treated in wild-type BT474 cells transfected with empty-vector (mock), BT474_USP32KO, and BT474KO_ReconWT cells for 24 h. The percentage of the sub-G1 population was measured by flow cytometry. Data are presented as mean and standard deviation of three independent experiments.

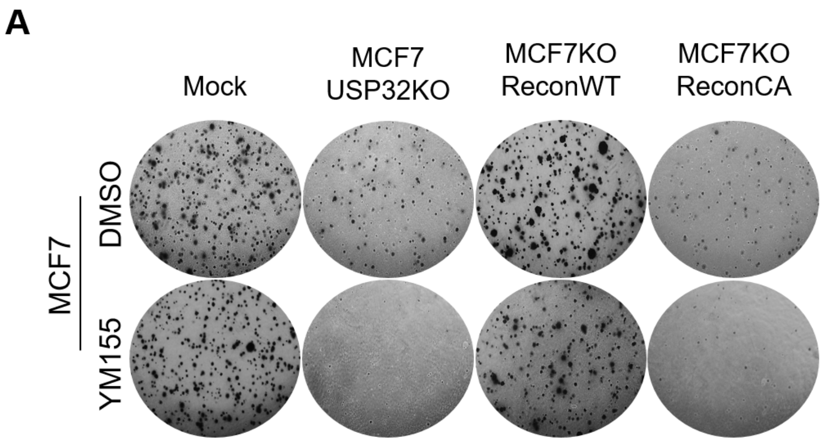

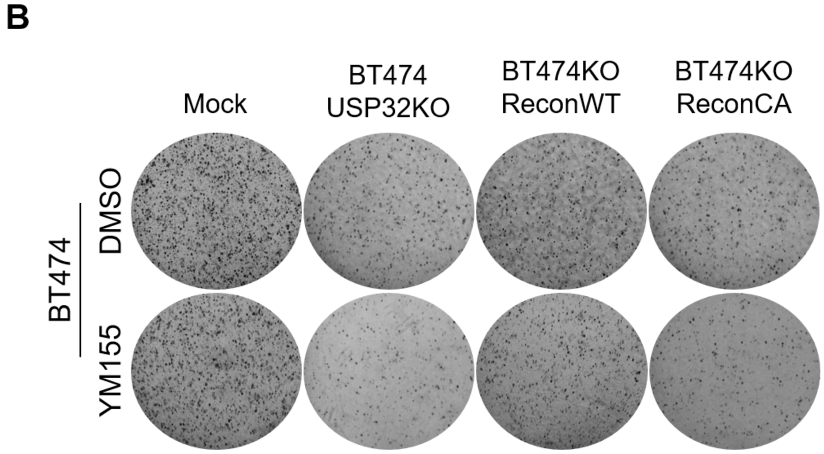

Figure S6. Colony formation assay in MCF7 and BT474 cells. (A) Mock MCF7, MCF7_USP32KO, MCF7KO_ReconWT, and MCF7KO_ReconCA cells treated with either DMSO or 5 nM YM155 for 14 days. **(B)** Mock BT474, BT474_USP32KO, BT474KO_ReconWT, and BT474KO_ReconCA cells were treated with either DMSO or 5 nM YM155 for 14 days. The colonies were stained with crystal violet for visualization. Assays were performed in triplicate and representative images are presented.

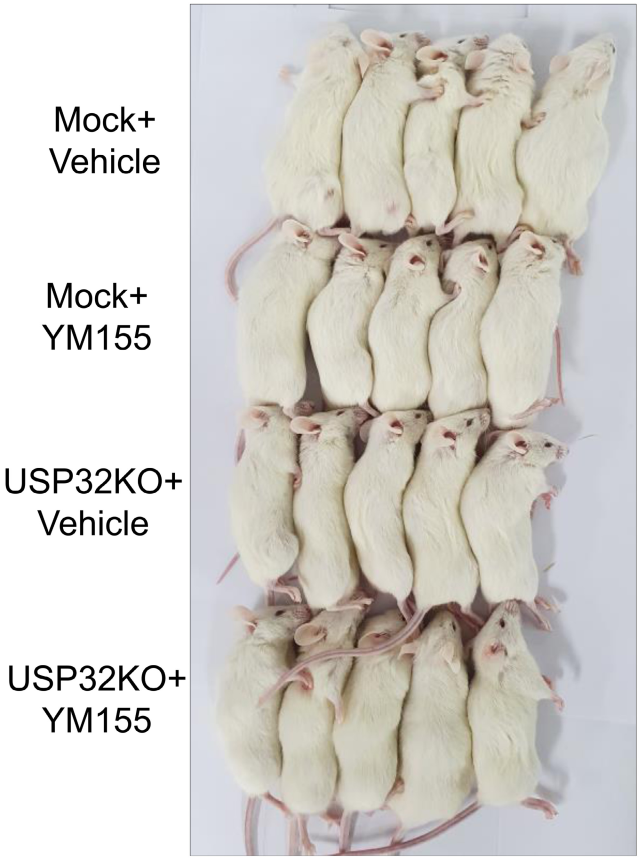

Figure S7. Mice carrying tumors in their right flanks at day 37.
